# The Effect of Dopaminergic Replacement Therapy on Creative Thinking and Insight Problem-Solving in Parkinson's Disease Patients

**DOI:** 10.3389/fpsyg.2021.646448

**Published:** 2021-03-05

**Authors:** Carola Salvi, Emily K. Leiker, Beatrix Baricca, Maria A. Molinari, Roberto Eleopra, Paolo F. Nichelli, Jordan Grafman, Joseph E. Dunsmoor

**Affiliations:** ^1^Department of Psychiatry, University of Texas at Austin, Austin, TX, United States; ^2^Neurology Clinic, Department of Neuroscience, Ospedale Civile S. Agostino Estense, Modena University Hospital, University of Modena and Reggio Emilia, Modena, Italy; ^3^Department of Psychology, University of Bologna, Bologna, Italy; ^4^Movement Disorders Unit at the IRCCS “Carlo Besta” Neurological Institute of Milan, Milan, Italy; ^5^Shirley Ryan AbilityLab, Chicago, IL, United States; ^6^Department of Physical Medicine and Rehabilitation, Neurology, Cognitive Neurology, Alzheimer's Center, Feinberg School of Medicine, Northwestern University, Chicago, IL, United States; ^7^Department of Psychiatry, Feinberg School of Medicine, Northwestern University, Chicago, IL, United States

**Keywords:** creativity, problem solving, insight, Parkinsion's disease, dopamine

## Abstract

Parkinson's disease (PD) patients receiving dopaminergic treatment may experience bursts of creativity. Although this phenomenon is sometimes recognized among patients and their clinicians, the association between dopamine replacement therapy (DRT) in PD patients and creativity remains underexplored. It is unclear, for instance, whether DRT affects creativity through convergent or divergent thinking, idea generation, or a general lack of inhibition. It is also unclear whether DRT only augments pre-existing creative attributes or generates creativity *de novo*. Here, we tested a group of PD patients when “on” and “off” dopaminergic treatment on a series of tests of creative problem-solving (Alternative Uses Task, Compound Remote Associates, Rebus Puzzles), and related their performance to a group of matched healthy controls as well as to their pre-PD creative skills and measures of inhibition/impulsivity. Results did not provide strong evidence that DRT improved creative thinking in PD patients. Rather, PD patients “on” medication showed less flexibility in divergent thinking, generated fewer ideas *via* insight, and showed worse performance in convergent thinking overall (by making more errors) than healthy controls. Pre-PD creative skills predicted enhanced flexibility and fluency in divergent thinking when PD patients were “on” medication. However, results on convergent thinking were mixed. Finally, PD patients who exhibited deficits in a measure of inhibitory control showed weaker convergent thinking while “on” medication, supporting previous evidence on the importance of inhibitory control in creative problem-solving. Altogether, results do not support the hypothesis that DRT promotes creative thinking in PD. We speculate that bursts of artistic production in PD are perhaps conflated with creativity due to lay conceptions of creativity (i.e., an art-bias).

## Introduction

Parkinson's disease (PD) is a degenerative disorder characterized by the loss of dopamine (DA) neurons in the bilateral nigrostriatal pathway (e.g., Deumens et al., 2002). This pathway connects the substantia nigra pars compacta with the dorsal striatum (putamen-caudate complex) leading to reduced function and the characteristic symptoms of tremor and bradykinesia (Gröger et al., 2014). Dopamine replacement therapy (DRT) is a widely used and effective treatment for PD which significantly improves motor symptoms. Physicians and doctors specialized in PD often report the emergence of a creative “talent” in PD patients after starting dopaminergic treatment. While this phenomenon is well-known among PD experts (Schrag and Trimble, 2001; Walker et al., 2006), it remains scientifically underexplored. Some empirical studies have examined the rise in creativity associated with DRT in PD patients; however most reports have been observational single-case studies or relied on limited measures of creativity such as a limited focus on divergent thinking (e.g., Walker et al., 2006; Canesi et al., 2012, 2016, 2017; Lhommée et al., 2014). Here, we attempt to shed light on the observation of enhanced creativity in PD patients on DRT by adopting an experimental approach (a test re-test and match control group comparison) and a comprehensive battery of creativity (including divergent thinking, convergent thinking, and idea generation).

Much of the evidence for an association between dopaminergic treatment in PD and enhanced creativity is from case reports. For example, Schrag and Trimble (2001) described the case of a patient diagnosed with PD who 4 years after the diagnosis started treatment with L-DOPA. Over the 1st month of therapy, he began writing poetry for the first time in his life. He had never written poetry before nor felt the desire to do so, yet his grandfather had written poetries and he was related to a well-known Irish poet. Schrag and Trimble speculated that the effect of dopaminergic and serotonergic drugs, either through cognitive enhancement, increased perception or a hypomanic syndrome in addition to selective fronto-cortical dysfunction, led to the release of previously inhibited creative power in this patient (Schrag and Trimble, 2001).

Another report described a patient with “artistic tendencies” for sketching that predated his PD diagnosis but increased after beginning DRT, where he began producing numerous pastel drawings a week—sometimes two a day (Walker et al., 2006). He was convinced that the medication increased his creativity and was unwilling to make dosage changes. Soon, unrelated to any change in medication, he also started presenting hypersexual disinhibition described as excessive flirting with women and asking them to pose nude for his artwork. Art critiques of the patient's works were varied. His work has been described as original with a strong sense of color and kinesthesia. Other critiques referred to it as naive and unoriginal, yet the output was so voluminous that some drawings were likely to be artistically successful. Pragmatically, his pieces had been shown in some local galleries, and he reported having sold over $2,000 worth of work in 1.5 years (Walker et al., 2006).

Another paper reported the case of a 68-year-old graphic designer affected by PD who was encouraged to paint and draw to fight post-PD depression (Chatterjee et al., 2006). At the beginning of DRT, his initial art was described as more representational, inspired by painters such as Van Gogh. Over time, with continued DRT, he began using colored pencils and his compositions became more abstract. He felt a strong urgency to produce as if “the train has left the station and I have just been served a delicious dinner in the cafe car. The train is picking up speed so I have to eat fast so I can finish my meal before we get to the last stop and I have to get off” (Chatterjee et al., 2006).

These intriguing case reports suggest that DRT might increase artistic “talent” in PD patients. A caveat to these reports, however, is that each patient possessed artistic tendencies before PD or was genetically related to someone with some artistic skills. Thus, these bursts of artistic output following DRT may facilitate creativity but may not “generate” it. Rather, underlying creative talent may be released or “burst out” by DRT.

Scientific and philosophical conceptualizations of creativity define it as the ability to produce responses that are both novel (i.e., original, rare, and unexpected) and suitable (i.e., adaptive and useful according to task constraints) regardless of its artistic properties (Sternberg and Lubart, 1996). Although case studies focus on “artistic-like” behaviors, such as writing poems and drawing, it is unclear whether they capture the components of novelty and originality that are considered essential to defining a product as creative. Artistic production has long been conflated with creativity (Sternberg and Lubart, 1996); this misperception, known as an “art bias,” is pervasive such that labeling someone as creative is typically reserved only for those with artistic talent (Runco, 2014). Thus, a key question remains whether DRT is associated with changes in truly creative artistic activities or merely compulsive artistic production, akin to the other forms of (often maladaptive) compulsive behaviors associated with DRT. Experimental examinations of creativity may be better suited to answering this question, using validated cognitive tasks to measure different types of creative thinking rather than relying on subjective assessments of creativity in patients' artistic products.

Creativity can be scientifically measured using tasks of divergent or convergent thinking and idea generation. Divergent thinking tasks consist of generating as many answers as possible to a specific question (e.g., “think of as many uses as you can for a brick;” Alternative Uses Task, AUT, Guilford, 1967). Convergent thinking tasks require participants to generate a single solution to a close-ended problem. An exemplary convergent thinking task for creativity is the Compound Remote Associates test (CRA), which assesses peoples' ability to identify associations among remote compound words (Bowden and Jung-Beeman, 2003). These problems are also used to study the cognitive processes of idea generation, as they can be solved either via sudden insight (i.e., “Aha!” moment) or in a step-by-step (i.e., analytic) fashion (e.g., Jung-Beeman et al., 2004). Many ideas or solutions to problems are generated through sudden insight that is then construed as creative thinking (Friedman and Förster, 2005; Salvi et al., 2018). Creativity is the result of multiple interacting cognitive processes supported by a large network of brain areas (Eysenck, 1993; Heilman, 2005; Beaty et al., 2016, 2018, 2019, 2020). A more thorough examination of divergent thinking, convergent thinking, and idea generation should help us understand whether DRT influences creative cognition and what aspects of creative thinking are impacted. Such an examination will also further advance the current understanding of DA function in creativity, PD, and DRT to inform neurobiological models of creativity.

Only a few studies experimentally investigated this rise of creativity in PD patients. Canesi et al. (2012) were among the first investigating the “artistic-like” production in PD by administering to a sample of 36 patients (with and without increased artistic-like production) and 36 healthy controls a validated divergent thinking creativity scale: the Torrance Test of Creative Thinking (Torrance, 1974). They found no difference between the creativity scores of the healthy controls and the PD patients with “artistic-like” behaviors. Also, PD patients without “artistic-like” behaviors scored less on elaboration compared to healthy controls and the other group of PD patients. Critically, PD patients increased their “artistic-like” behaviors after starting DRT and spent most of the day pursuing their newly acquired artistic interests, disregarding their social life and daily duties—a feature that could resemble compulsive behavior and punding (Fasano and Petrovic, 2010). The authors reasoned that if the creativity expressed by PD patients is part of DRT then more creative patients should present an attitude for impaired impulse control. However, there was no evidence of increased impulsivity or deficits in impulse control among the PD patients who reported an increase in the creative drive after starting DRT. The same research group in 2016 evaluated whether the artistic production and creativity of PD patients are influenced by DRT or linked to “artistic-like” skills, in two groups of PD patients who were professional artists and non-artists, and two groups of matched healthy controls of professional artists and non-artists. Their results found the creativity score was significantly higher in the two groups of artists (PD and control) than in the other groups, and there was no difference in impulse control disorders between PD groups. By contrast, Faust-Socher et al. (2014) tested a group of PD patients treated with DRT on divergent thinking (measured using the Tel Aviv University Creativity Test-Visual, Milgram and Milgram, 1976), convergent thinking (measured using the Remote Associates Test; Mednick, 1968) and novel metaphors production. They found that PD patients had enhanced divergent (but not convergent) thinking and comprehension of novel metaphors and compared to neurologically healthy controls and that these features were unrelated to impulse control disorder (measured using the QUIP-RS, Weintraub et al., 2012). The authors speculated that DRT might lead to a reduction in latent inhibition, resulting in broadening the associative network and thus enhanced creativity, leaving the issue of the role of inhibition open once more.

Although prior studies have not found evidence of a link between DRT-related changes in creativity and impulse control disorder symptoms in PD patients, task-based measures of inhibitory control might provide a more subtle indication for inhibitory control deficits that, while not rising to the level of impulse control disorder, might contribute to changes in creative cognition or trigger a compulsive artistic-like behavior that might be confused as creative. Indeed, if DRT is not well-regulated, it can result in impulse control disorders, a serious complication that comprises compulsive gambling, shopping, sexual behavior, hobbyism, hoarding—and punding— a stereotyped behavior characterized by an intense fascination with a complex, excessive, nongoal oriented, and repetitive activity (Fasano and Petrovic, 2010). However, creativity is positively related to executive functioning, specifically working memory updating and inhibition (Benedek et al., 2014) allowing us to hypothesize that worse cognitive control in PD patients should negatively predict creativity. From the literature, we know that overall PD patients have impaired executive functions, including inhibitory control or working memory updating (e.g., Hsieh et al., 2008), and late-onset PD is associated with augmented Stroop interference (Henik et al., 1993). Specifically, Brück et al. (2005) found that greater Stroop interference in PD patients is related to reduced L-dopa uptake in the medial frontal cortex and anterior cingulate, brain areas which are also involved in creativity and insight problem-solving (e.g., Subramaniam et al., 2009; Beaty et al., 2016). This result allows us to predict a negative relation between inhibition and creativity in PD patients.

In sum, the scientific literature on the emergence of presumed creativity in PD patients undergoing DRT is sparse, and further experimental evidence and replicability are needed to support a hypothesis that DRT promotes creativity in PD. The few experimental studies that have examined this topic employed different scales to assess creativity and focused on different aspects, making results difficult to compare across studies. The extant literature indicates that PD patients seem to develop an artistic “talent” only after they begin DRT. However, existing research does not address whether this behavior meets operational definitions of creativity from the scientific literature (e.g., presenting real components of novelty and originality) or if it is simply an “artistic-like” behavior as defined by Canesi et al. (2012). To shed light on this matter, one avenue is to measure how increased DA levels impact the different components of creativity: divergent and convergent thinking or idea generation itself (i.e., insight vs. step-by-step). Therefore, we administered several different task-based creativity measures to specifically assess divergent and convergent thinking as well as idea generation. This test-retest design allows us to evaluate the within-subject effects of DA medication on the different components of creativity; this is particularly important considering there are well-known individual differences in these measures. We hypothesize that if DRT enhances creativity, we should find a significant difference within the same patients when “off” compared to when “on” medication in at least one of the different components of creativity: divergent and convergent thinking or idea generation. Additionally, we hypothesize that if DRT plays a role in people's creativity, we should find a significant difference between PD patients when “on” medication vs. a control group of adults at the same age and level of education without PD who are not taking a dopamine agonist.

“Artistic-like” behaviors decrease significantly following a reduction in DA treatment, leading to the conclusion that this effect is at least partially related to DA drug administration. However, research on creative and non-creative PD patients shows that this “skill” is more likely present in patients who were already creative, or perhaps patients who are genetically related to an artist. Thus, it will be important to determine what creative predispositions PD patients may have expressed prior to their PD diagnosis, to contextualize any potential observed changes in creative behaviors emerging after DRT. In other words, understanding any artistic predispositions will help us evaluate whether any observed changes in creativity is an “awakening” or “reawakening” of creativity. To better understand this relation, we assessed lifetime creative achievement (Creative Achievement Questionnaire; Scott et al., 2005), hobbyism and artistic-like punding behaviors (Hobbyism and Artistic-like Behaviors Punding Scale), and whether the patient considers themselves a creative or analytical person (see methods for further description).

The rising of “artistic-like” behaviors is often associated with increased DRT, which manifests with a lack of inhibition leading to hypersexuality, gambling, or punding. One hypothesis is that increased DA levels may disrupt inhibition (Chakravarty, 2010) by altering DA pathways involved in the modulation of reward, motivation, and inhibitory control (Kulisevsky et al., 2009). Reduced inhibition is thought to be associated with PD and with creativity; therefore, the general hypothesis is that DRT may reduce inhibitory control through the stimulation of these pathways (Antonini and Cilia, 2009), possibly affecting creativity. However, the “artistic-like” behavior could be merely related to compulsive punding (Fasano and Petrovic, 2010) and lack of inhibition with no real component of creativity. Considering the relationship between DA and creativity, in this study we investigate the relation between inhibition and creativity in PD patients, and test if the increase of DA associated with DRT enhances creativity in PD patients “on” and “off” medication, or if the “artistic-like” behaviors commonly seen in PD patients on these medications are merely a compulsive reaction to the drugs.

## Methods

### Subjects

Thirteen Parkinson patients (PD group: four females; average age = 56.5 ± 9 years) and 26 healthy controls (HCs: 15 females; average age = 61.3 ± 7 years) participated in the study. The experiment took place at the Neurologic Clinic of the University of Modena and Reggio Emilia, and at the Unit for Parkinson's Disease and Movement Disorders of the Fondazione I.R.C.C.S. Istituto Neurologico Carlo Besta, Milano. The PD group was recruited from a group of PD patients set to undergo two sessions of standard clinical assessment before receiving deep brain stimulation surgery: once while “on” regular DRT (treated with a stable DRT for at least 4 months prior to their neurological and neuropsychological evaluation) and another “off” medication (i.e., following overnight withdrawal from DRT). All PD patients were right-handed and met the clinical diagnostic criteria for PD measured according to the UK Brain Bank criteria (Hughes et al., 1992), without comorbid dementia (as determined via neuropsychological examination, see material). HCs were recruited from the general population, were all right-handed, and were matched to the PD patients on age and years of education.

Each PD patient was tested while “on” and “off” DRT medication in separate experiment sessions within a two-week interval (M = 2 ± 3.4 days, range = 0–13 days). PD patients were randomly assigned to one of two test-retest orders (ON-OFF: *n* = 6, OFF-ON: *n* = 7) to counterbalance medication status order across participants and control for potential order effects. Two PD patients exhibited rapid motor movement problems and were pre-assigned to the OFF-ON order to complete the test-retest sessions in a single day (i.e., tested in the morning and afternoon). All other PD patients completed testing on separate days, within 1–3 days for the majority of the group (~77%). HCs also completed test-retest sessions on separate days, with the majority (~92%) completing testing within a two-week interval (M = 5.6 ± 4.3 days, range = 1–14 days). Two participants fell outside this interval and were excluded from analysis, resulting in a final group of 24 HCs (13 females; average age = 61.5 ± 7 years).

This study was approved by the Ethical Committee of the Area Vasta Emilia-Nord, and all participants provided written informed consent prior to testing.

### Material

**Neuropsychological Assessments**. All participants were administered a standard cognitive neuropsychological assessment battery to screen for dementia and evaluate various aspects of cognitive function (including executive function, memory, language ability, etc.). Specifically, they were administered the Mini-Mental State Examination (MMSE; Folstein et al., 1975), Raven's Progressive Matrices (RPM; Raven, 1965), Stroop Test (ST; Stroop, 1935), Forwards and Backwards Digit Span (DS-F, DS-B; Wechsler Abbreviated Scale of Intelligence, 1999), Free and Cued Selective Reminding Test with Immediate and Delayed Recall (FCSRT-IR; Grober and Buschke, 1987), Phonemic and Semantic Verbal Fluency Tests (VFT-F, VFT-S; Costa et al., 2014), and Frontal Assessment Battery (FAB; Dubois et al., 2000).

**Creative Achievement Questionnaire (CAQ**) assesses creative achievement across 10 domains, including visual arts, music, dance, architectural design, creative writing, humor, inventions, scientific discovery, theater, and film, and culinary arts (Ludwig, 1992). Each domain consists of eight items describing different forms of achievement with different levels of notoriety. For example, in the Music domain, items range from “I have no training or recognized talent in this area” to “My compositions have been critiqued in a national publication.” Total scores were computed by summing across the 10 domains for each participant (see Carson et al., 2005).

**Barratt Impulsiveness Scale (BIS-11A)** measures impulsive personality traits (Fossati et al., 2001). It includes 30 items describing different forms or degrees of impulsivity that are scored as six first-order factors and three second-order factors, reflecting subdomains of impulsiveness: (1) attention impulsivity and cognitive instability (attentional domain); (2) motor impulsivity and perseverance (motor domain); and (3) self-control and cognitive complexity (non-planning domain (Patton et al., 1995). For each item, participants rate how frequently they display the described behavior using a 4-point scale. Total scores were computed by summing across all items for each participant (Patton et al., 1995).

The **QUIP-Rating Scale (QUIP-ICD and RS)** measures symptoms for impulse control disorders (Weintraub et al., 2012), and changes in symptom severity over time (Weintraub et al., 2012). The QUIP-RS includes questions assessing the frequency of behaviors in the previous 4 weeks (or another 4-week timeframe) associated with each of 7 ICDs and related disorders, including compulsive gambling, sexual behavior, buying, eating, hobbyism, punding, and medication use. Participants rate each item using a 5-point scale ranging from never to very often, and items are scored according to increasing frequency (i.e., 0 = never, 1 = rarely, 2 = sometimes, 3 = often, 4 = very often). Total scores were computed by summing across all items for each participant.

**Hobbyism and Artistic-like Behaviors Punding Scale (HABPS)**. Punding is a repetitive and stereotyped behavior defined by an intense fascination with complex, excessive, and repetitive activities that are often non-goal oriented (Fasano and Petrovic, 2010). Activities representative of such behavior can include repetitive tinkering with technical equipment like radio sets, watches, or car engines, or compulsive sorting, tidying, hair-brushing, or nail-polishing. “Punders” tend to be aware of the excessive nature of their behavior yet are often unable to stop it. These behaviors are commonly observed following high levels of DRT medication administered to Parkinson's patients, as well as cocaine and amphetamine use in addicts (Fasano and Petrovic, 2010). In this study, we created a modified version of the Punding Rating Scale (adapted from Fasano and Petrovic, 2010) to deeply investigate the nature of these hobbies and their “artistic-like” components. The complete scale is provided in the Supplementary Materials.

### Divergent Thinking

The *Alternative Uses Task* (AUT) is an established measure of divergent thinking (Guilford, 1967). In this task, participants are asked to list as many possible uses of a common object item (i.e., a brick). A total of six common objects (spoon, brick, newspaper, rubber band, sheet, and sock) were administered across the test-retest sessions, resulting in three objects per session. We adopted a subjective scoring system, using raters blind to participants' group assignment, experimental conditions order of the responses (Hass and Beaty, 2018; Hass et al., 2018). The following four score components were calculated for analysis as follows: *Fluency*: the number of distinct uses per item; *Flexibility*: the number of distinct categories of uses per item; *Elaboration*: the number of details in the description given for each use of the item (e.g., “a doorstop” counts as 0, whereas “a doorstop to prevent a door slamming shut in a strong wind” counts as 2); *Originality*: the number of unique or uncommon uses generated by a participant, out of the full range of responses from all participants [see Hass and Beaty (2018) and Hass et al. (2018) for the reliability of the scoring methods and subjective rating].

### Convergent Thinking

*Compound Remote Associate (CRA)* problems were developed to study creativity without relying on domain-specific knowledge [originally by Mednick (1968) and Bowden and Jung-Beeman (2003)]. It consists of a set of problems, each composed of three cue words associated with a fourth solution word that the participant must generate. Each cue word may combine with the solution word to form a compound word or phrase or may relate to the solution word by semantic association or synonymity (e.g., problem: tooth/potato/heart — solution: SWEET). The example used in the Italian language was *scuola, tutto*, and *domani* (translation: school, all, tomorrow) which forms the compound *doposcuola, dopotutto, and dopodomani* (translation: afterschool, after all, after tomorrow; Salvi et al., 2015b, 2018). For this study, CRA problems consisted of 40 trials per session (for a total of 120 Italian CRA problems, taken from Salvi et al., 2015b). The CRA problems have been used specifically for investigating the neural bases of insight problem-solving (see e.g., Jung-Beeman et al., 2004; Bowden et al., 2005; Kounios et al., 2006; Salvi et al., 2015a, 2020a,c; Sprugnoli et al., 2017; Santarnecchi et al., 2019; Becker et al., 2020b) and other cognitive and social factors associated with it (e.g., Salvi et al., 2016a,b, 2020b; Cristofori et al., 2018; Salvi and Bowden, 2019; Threadgold et al., 2019).

*Rebus Puzzles* involve a combination of visual, spatial, verbal, or numerical cues from which a set of principles are used to encrypt a phrase or saying that is well-known to participants (MacGregor and Cunningham, 2008). These puzzles require overcoming learned grammar rules of word composition to reconstruct the meanings of words. To solve each Rebus Puzzle, participants have to restructure the formal interpretation of reading by relaxing their ingrained constraints, to shift how the problem elements are perceptually or cognitively represented. A common way to solve Rebus Puzzles is to verbally interpret the visual-spatial connections of the problem components (e.g., location, font size, style, color, or the spacing between the letters or words) and incorporate them into the solution. The Rebus Puzzles used in this study consisted of 30 trials per session [selected out of a total set of 88 Italian Rebus Puzzles developed by Salvi et al. (2015b)] that presented a word or words in an informative pictorial fashion, from which individuals derive a common expression (e.g., *CYCLO CYCLO CYCLO* is solved as “*tricycle*,” because CYCLO is written three times). These puzzles have the advantage of being relatively easy to present and having well-constrained situations, and have been used specifically for investigating insight problem-solving and cognitive and social factors associated with it (e.g., MacGregor and Cunningham, 2008; Threadgold et al., 2018; Salvi et al., 2020b).

CRAs and Rebus Puzzles are hybrid-type convergent thinking problems that can be solved through insight or step-by-step processes of idea generation, with participants on each successful trial required to report which of the two most contributed to problem-solving. Self-reports differentiating between insight and step-by-step problem-solving are reliable, and behavioral and neuroimaging markers have robustly demonstrated that the reports reflect distinct cognitive processing (e.g., Jung-Beeman et al., 2004; Salvi et al., 2015a, 2020a,c; Becker et al., 2018, 2020a; Santarnecchi et al., 2019).

Performance on the CRA and Rebus Puzzles was scored for: (1) *accuracy*: the proportion of correctly solved trials; and the rate of (2) *commission errors*: the proportion of trials solved incorrectly; (3) *omission errors (i.e. timeouts)*: the proportion of trials where time ran out before a solution was reached; (4) *solutions via insight*: the proportion of problems solved correctly through insight; (5) *solutions via step-by-step*: the proportion of problems solved correctly through step-by-step. All proportions included the total number of task trials given as denominator.

### Procedure

Prior to the first testing session, each participant underwent a standard cognitive neuropsychological assessment battery to screen for dementia and evaluate general mental. These tests were part of the clinical screening for the PD patients' assessment. Following the initial neuropsychological evaluation, participants were administered the creativity tasks.

The creativity tasks were administered to PD patients and HCs in two balanced sessions. PD patients were tested “on” and “off” DRT medication.

For the divergent thinking task, participants completed one block of 3 AUT objects per session. Participants were instructed to list as many possible uses as they could for each of the three common household objects within the allotted time (~3 mins per object). Participants were told to verbally announce all responses for the Experimenter to record. The order of objects was randomized within and between sessions.

For the convergent thinking tasks, participants completed one block each of CRA problems and Rebus Puzzles per session for ~30–40 and 20–30 min, respectively. The task blocks were ordered in an ABBAAB or BAABBA counterbalanced fashion across problem types (i.e., CRA and Rebus Puzzles) and between different blocks of the same problem type (i.e., across sessions). Participants received verbal and written instructions for the CRA and Rebus Puzzles before starting the experiment and were instructed on how to judge each solution process as insight vs. step-by-step^1^. They first went through one example and six practice trials in which they made the ratings and demonstrated that they understood the task. It was explained to participants that neither insight nor step-by-step were the “correct” way to attain the solution, rather they were being asked whether they used insight-like or step-by-step-like problem-solving to reach their solution. The experiment began only once participants were familiar with the task and could clearly distinguish when a solution was reached via insight or step-by-step.

Each trial started with a 1s fixation cross followed by the presentation of a READY prompt and followed by a second 1s fixation cross. Afterward, the problems were presented on the screen one-at-a-time. CRA and Rebus Puzzles were presented centrally in black font on a white background, with CRA cue words presented in standard horizontal orientation at, above, and below the center of the screen. All the participants had 20 s to solve each problem and were instructed to verbally announce the solution as soon as they attained it for the Experimenter to record. No feedback was given regarding the accuracy of the solution. Once the participant said the potential solution, the Experimenter pressed the spacebar on the keyboard. This was done to mitigate the potential impact of speed and motor difficulties on response rates and timeouts for PD participants. Following the production of each solution, participants indicated whether the problem was solved through insight or step-by-step to the Experimenter who recorded their response. Self-reports differentiating insight and step-by-step problem-solving are reliable and associated with several distinct behavioral and neuroimaging markers (Bowden and Jung-Beeman, 2003; Jung-Beeman et al., 2004; Subramaniam et al., 2009; Salvi et al., 2015a, 2020a,c) first went through one example and). The experimental procedure was presented using E-Prime 2.10 on a laptop at a viewing distance of about 60 cm.

## Results

### Creative Performance

**Figure 1:**
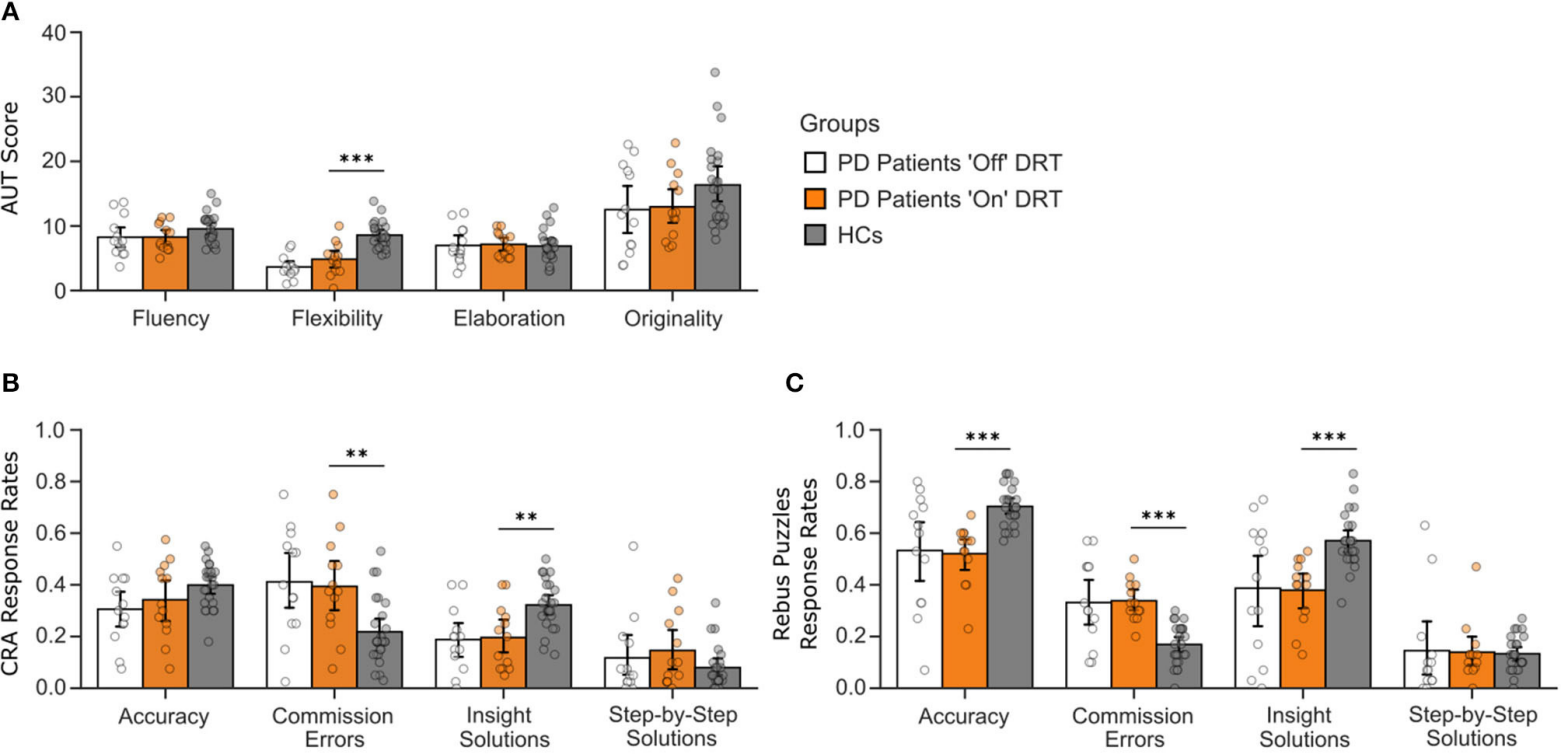
Divergent and convergent thinking performance of PD patients “on” and “off” DRT compared to HCs. **(A)** Performance of PD patients “on” and “off” DRT medication compared to HCs on four measures of divergent thinking. **(B,C)** Performance of PD patients “on” and “off” DRT medication compared to HCs on convergent thinking measured with the CRA and Rebus puzzles tasks. DRT, Dopamine Replacement Therapy; PD, Parkinson's Disease; HCs, Healthy Controls; AUT, Alternate Uses Test; CRA, Compound Remote Associates task. **BF_10_ between 1 and 3, ***BF^10^ > 3.

**Figure 2:**
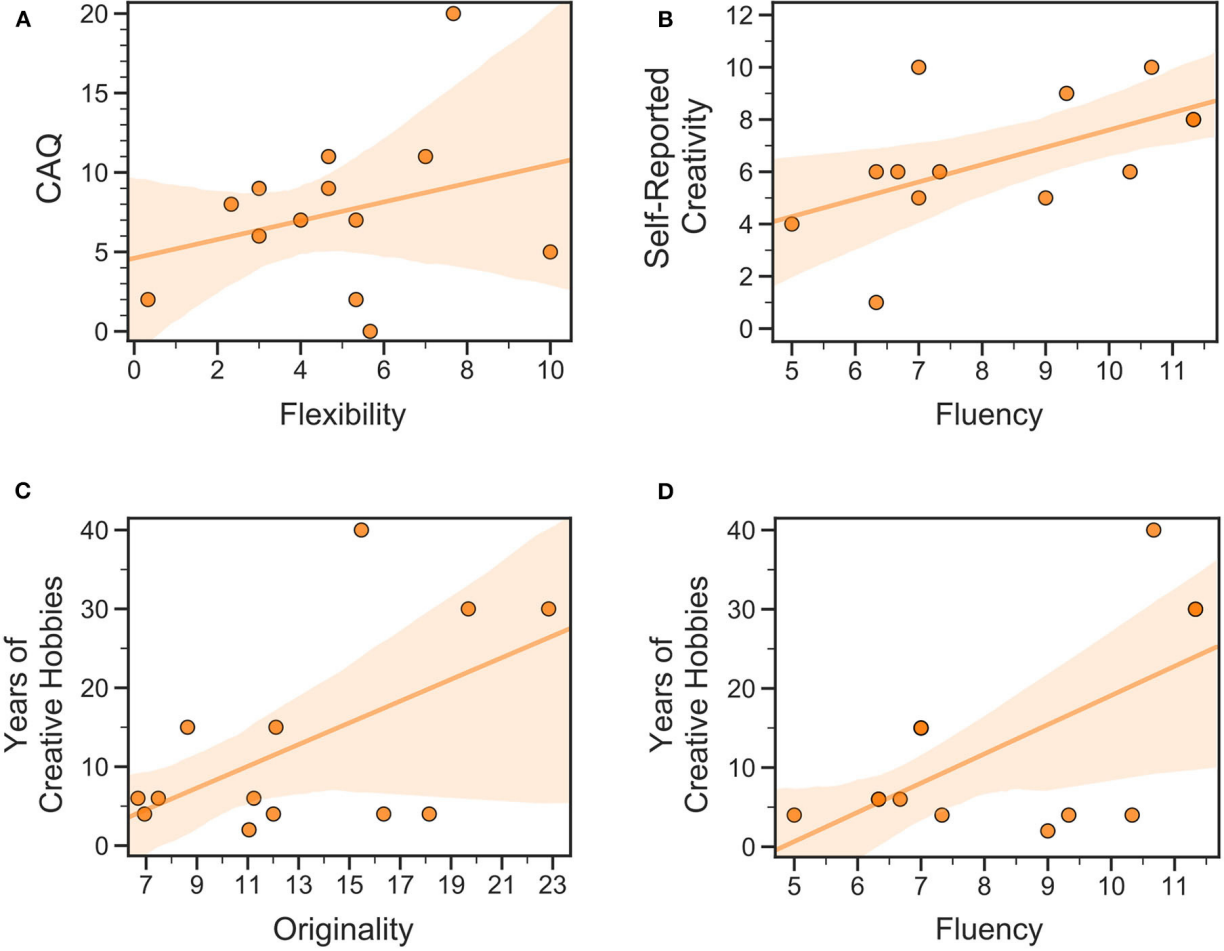
Linear regression models of real-life creativity predicting effects of DRT on divergent thinking for PD patients “on” medication. Models showed significant positive relations between **(A)** CAQ and flexibility; **(B)** self-reported creativity and fluency; **(C)** years of creative hobbies and originality; and **(D)** years of creative hobbies and fluency. CAQ, Creative Achievement Questionnaire.

**Figure 3:**
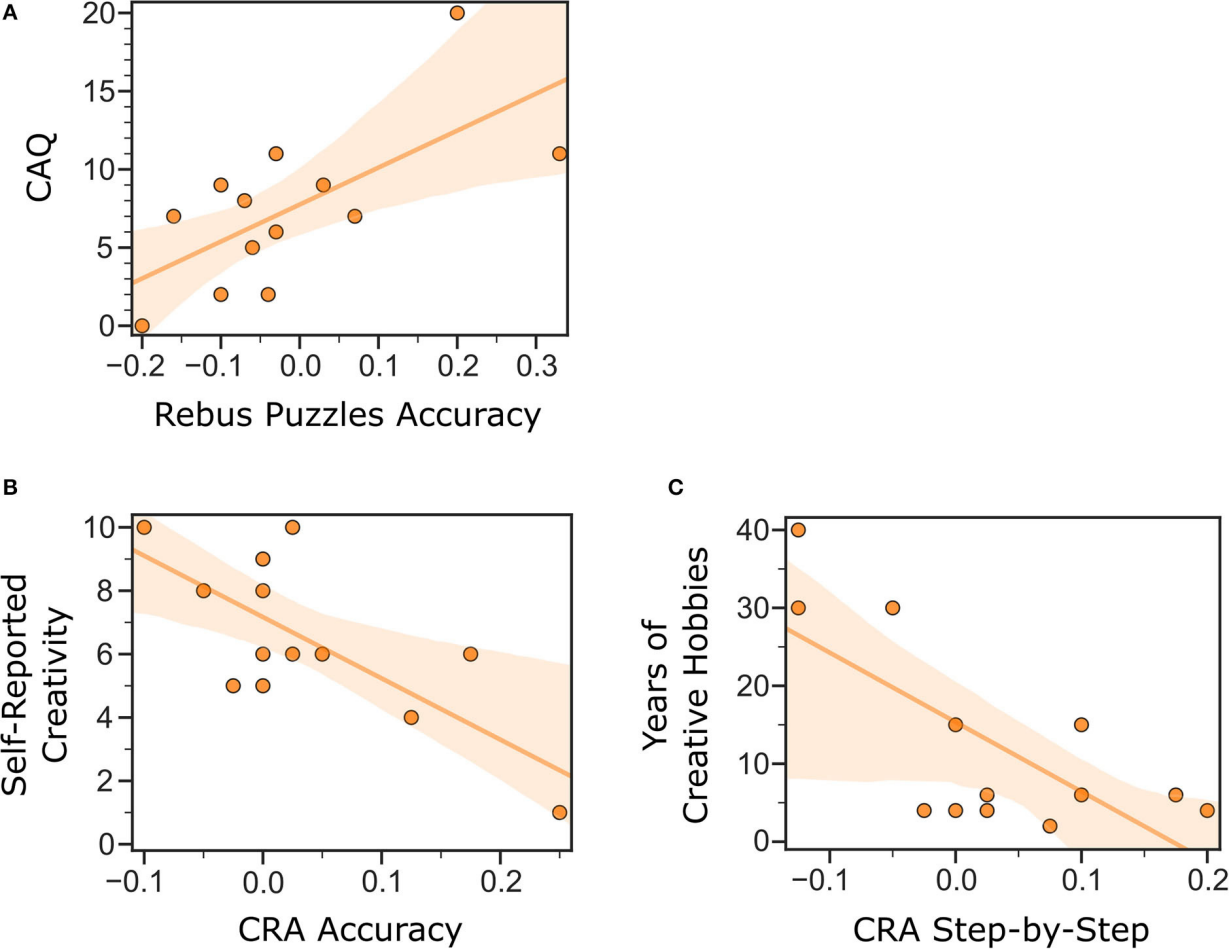
Linear regression models of pre-existing real-life creativity predicting effects of DRT on convergent thinking for PD patients “on”-“off” medication. Models showed a significant: **(A)** positive relation between CAQ and Rebus Puzzles accuracy. **(B)** Negative relation between self-reported creativity and CRA accuracy. **(C)** Negative relation between years of creative hobbies and solutions *via* step-by-step. CAQ, Creative Achievement Questionnaire; CRA, Compound Remote Associates Task.

To investigate if DRT enhances creativity, we compared divergent and convergent thinking and idea generation performance within PD patients “on” vs. “off” DRT and compared performance from PD patients “on” medication to healthy adults. Descriptive statistics and results for the group comparisons are summarized in Supplementary Tables 1–3. Significant findings are shown in Figures 1–3.

Performance on the AUT served as the primary measure of divergent thinking. Paired sample *t*-tests showed no significant effects of DRT on PD patients' AUT performance “on” vs. “off” medication for fluency, flexibility, elaboration, or originality (BF_10_ = 0.28–0.92, indicating mild to moderate evidence in favor of H_0_)^2^. Independent sample *t*-tests on divergent thinking in PD patients “on” DRT vs. HCs showed significantly lower flexibility on the AUT for PD patients (BF_10_ = 626.1, indicating strong support for H_1_). There were no other significant group differences (BF_10_ = 0.34–0.91; see Figure 1A and Supplementary Table 2 for details).

No significant effects of DRT on PD patients' performance “on” vs. “off” medication were found (BF_10_ = 0.28–0.61). Independent sample *t*-tests on CRA performance revealed that PD patients “on” DRT solved significantly fewer problems *via* insight and made significantly more commission errors than HCs (BF_10_ = 19.7 and 17.5, respectively; see Figures 1B,C, and Supplementary Table 3 for details). PD patients “on” DRT solved significantly fewer Rebus Puzzles *via* insight, were less accurate, and made significantly more commission errors than HCs (BF_10_ = 670.6−26,589.5; see Supplementary Table 3 for details). There were no other significant group differences (BF_10_ = 0.34–1.09).

### Creative Predispositions

We also investigated whether measures of creativity were affected by creative predispositions in PD patients prior to their diagnosis. Because we recruited patients who were already diagnosed with PD, we considered creative achievements (CAQ total), Years of Creative Hobby (HABPS -Item 2), and Self-Reported Creativity (HABPS -Item 3) as different measures of real-life creativity. Effects of DRT on divergent and convergent thinking were indexed as the within-subject change in performance for PD patients “on” minus “off” medication for the AUT sub scores and for the accuracy and rate of problems solved *via* insight and step-by-step on the CRA and Rebus Puzzles.

To understand how real-life creativity might differentially relate to creative thinking in PD patients under DRT vs. HCs, we tested for group differences in the linear regression analysis of real-life and task-based creativity using Fisher's *z*-test for comparing independent correlations (Preacher, 2002). In this method, Fisher's *z*-transformation is applied to the correlation coefficients from each group, and the *z*-transformed correlation coefficients are tested for group differences using independent sample *t*-tests. See Figure 2 for details.

For PD patients “on” DRT, CAQ has a linear relationship with flexibility [*F*_(1, 11)_ = 6.35, *R*^2^ = 0.36, *p* = 0.028; BF_10_ = 3.0], years of creative hobbies with fluency [*F*_(1, 11)_ = 7.17, *R*^2^ = 0.39, *p* = 0.021; BF_10_ = 3.7] and originality [*F*_(1, 11)_ = 5.13, *R*^2^ = 0.31, *p* = 0.045; BF_10_ = 2.1], and self-reported creativity has a linear relationship with fluency [*F*_(1, 11)_ = 5.05, *R*^2^ = 0.31, *p* = 0.046; BF_10_ = 2.1].

The same analyses within HCs yielded no significant correlations (BF_10_ = 0.36–0.52).

Fisher's *z*-tests comparing the significant correlation coefficients from PD patients “on” DRT to HCs showed that PD patients “on” DRT had significantly higher positive correlations between years of creative hobbies and fluency (*z* = 2.86, *p* = 0.004) and originality (*z* = 2.32, *p* = 0.02). No other significant group differences emerged. See Figure 2 and Table 2 of Supplemental Materials for details.

CAQ has a linear relationship with the effects of DRT on Rebus Puzzles accuracy for PD patients “on” vs. “off” medication [*F*_(1, 11)_ = 8.97, *p* = 0.012; *R*^2^ = 0.45; BF_10_ = 5.8]. For the CRA, self-reported creativity has a negative linear relationship with the change in accuracy with DRT in PD patients [*F*_(1, 11)_ = 12.13, *p* = 0.005; *R*^2^= 0.53; BF_10_ = 11.6], and years of creative hobbies negatively predict the effects of DRT on the rate of step-by-step solutions for PD patients [*F*_(1, 11)_ = 11.19, *p* = 0.007; *R*^2^ = 0.50; BF_10_ = 9.5]. The equations for these significant regression models are as follows:

$$\begin{array}{l} \Delta \text{Rebus Puzzle Accuracy with DRT}=(0.02*\text{CAQ})-0.15\\ \Delta \text{CRA Accuracy with DRT}=(-0.03*\text{Self-Reported Creativity})\\ \;+0.21\\ \Delta \text{CRA Step-by-Step Solutions with DRT}\\ \;=(-0.01*\text{Years of Creative Hobbies})+0.10 \end{array}$$

No significant correlations were observed for real-life creativity and convergent thinking performance in PD patients “on” DRT or HCs (BF_10_ = 0.25–1.44); therefore, no group comparisons were tested. See Figure 3 and Table 3 of Supplemental Materials for details.

#### Art-Bias Hypothesis

As mentioned in the introduction, there is a lay conceptualization of creativity that rests entirely on “artistic-like” behaviors. This conceptualization of creativity may in large part explain the observation that PD patients who practice artistic hobbies are more creative on DRT. To test this hypothesis, we ran a linear regression analysis between the number of years patients were practicing a hobby and self-reported creativity. Results showed that in PD patients, years of hobbies have a linear relationship with self-reported creativity [*F*_(1, 11)_ = 5.42, *p* = 0.040; *R*^2^ = 0.33; BF_10_ = 2.3]. By contrast, CAQ was not related with self-reported creativity (BF_10_ = 0.37). Thus, the amount of time practicing a hobby influences PD patients' perception of their own creativity.

#### Inhibition in Creative Thinking Under DRT

One of our hypotheses aims to understand the role of inhibition on the emergence of creative “talent” in PD patients under DRT. To this end, we compared performance on several clinical assessments of impulsivity and inhibition (BIS Total, QUIP-RS, Stroop-EIT, and Stroop-EIE) with convergent and divergent thinking scores in PD patients. In general, worse Stroop interference (i.e., reduced inhibition) was significantly correlated with impaired convergent thinking in PD patients “on” DRT, but no significant correlations emerged for divergent thinking performance (BF_10_ = 0.35–0.77). Stroop EIE has a negative linear relationship with convergent thinking for the CRA [*F*_(1, 11)_ = 12.75, *p* = 0.004; *R*^2^ = 0.53; BF_10_ = 13.1], and Rebus Puzzles [*F*_(1, 11)_ = 14.92, *p* = 0.003; *R*^2^ = 0.57; BF_10_ = 19.7] accuracies. Stroop-EIT has a negative linear relationship with Rebus Puzzles accuracy for PD patients “on” DRT [*F*_(1, 11)_ = 6.88, *p* = 0.024; *R*^2^ = 0.38; BF_10_ = 3.4]. In addition, Stroop-EIT has a linear relationship with commission errors on the CRA [*F*_(1, 11)_ = 5.02, *p* = 0.047; *R*^2^ = 0.31; BF_10_ = 2.0] and has a negative linear relationship with step-by-step solution rate on the CRA for PD patients “on” DRT [*F*_(1, 11)_ = 6.63, *p* = 0.018; *R*^2^ = 0.31; BF_10_ = 2.2].

## Discussion

PD is mainly known as a movement disorder characterized by bradykinesia and tremor. There is also increasing interest in the cognitive aspects associated with this disease, such as the preservation, and even enhancement, of artistic skills in PD patients under DRT (e.g., Cools et al., 2001a,b; Inzelberg, 2013). The link between this neurodegenerative disease and an enhanced artistic production as an expression of creativity has been reported in several single case studies (e.g., Schrag and Trimble, 2001; Chatterjee et al., 2006; Walker et al., 2006). However, the experimental literature using objective measures of creative thinking remains scarce. It has been unclear if artistic output by PD patients on DRT reflects an objective enhancement in creativity defined by the features of novelty and originality or is simply in an increase in artistic production that meets lay conceptions of creativity. The role of pre-PD creative abilities has also remained largely unexamined. While some studies report that creative predispositions play an important role in the creative “talent” of PD patients on DRT, others do not support this conclusion (Chatterjee et al., 2006, Schrag and Trimble, 2001; Walker et al., 2006; Canesi et al., 2016). Because these results refer to only single-patient reports and a handful of unreplicated experimental studies, much remains unknown about bursts of putative creativity observed in some PD patients on DRT. The results reported here do not support the conclusion that DRT enhances objective measures of creativity in PD patients. We found no difference in divergent thinking measured with the AUT, in both of our convergent thinking measures (CRA and Rebus Puzzles) and idea generation (*via* insight or step-by-step) in PD patients while they were “on” vs. “off” medication. Importantly, even if patients presented some level of artistic tendencies or compulsive behaviors that involve artistic activities, we found no evidence suggesting that they become more creative on DRT. We also investigated whether PD patients under DRT showed better performance on creative thinking tasks than healthy controls of similar age and level of education. PD patients did not outperform matched controls on divergent thinking, further indicating that divergent thinking is not enhanced by DRT in PD patients. PD patient's performance was indeed worse than controls in flexibility, suggesting that cognitive flexibility impaired by PD is not ameliorated by DRT, at least within the context of creative problem-solving. This result corroborates Canesi et al.'s (2012) results that PD patients scored lower on flexibility than healthy controls on the Torrance Test of Creative Thinking. Therefore, artistic “talent” showed by PD patients on DRT does not translate into cognitive flexibility on objective measures of divergent thinking used to assess creative thinking.

Our results show a consistent difference in idea generation in both of our convergent thinking measures: the CRA and Rebus Puzzles. Specifically, PD patients “on” DRT solved a lower proportion of problems via insight and made more commission errors, compared to HCs. The fact that PD patients, even when “on” medication, showed impaired insight problem-solving abilities can be explained by the relation between insight and DA. Brain regions associated with insight-based problem-solving, such as the anterior cingulate cortex and the right superior temporal gyrus, have a high density of DA receptors (Tune et al., 1996; Jung-Beeman et al., 2004; Lumme et al., 2007; Aziz-Zadeh et al., 2009; Subramaniam et al., 2009; Salvi et al., 2020a). Additional evidence linking DA to insight comes from research using the eye-blink to index levels of striatal DA. Spontaneous eye-blink rate is considered a reliable indicator of DA activity in both PD patients and the general population (Karson, 1983; Blin et al., 1990; Kleven and Koek, 1996; Taylor et al., 1999; Colzato et al., 2007, 2009) and is often used as a proxy for striatal DA production (Karson, 1983; Taylor et al., 1999). Studies on eye-blink rate and creativity show an inverted *U*-shaped relationship between eye-blink rate and creativity as assessed by the AUT and the CRA (Akbari Chermahini and Hommel, 2010). Specifically, solutions *via* insight are associated with a higher eye-blink rate compared to solutions *via* step-by-step analysis (Salvi et al., 2015a). Such a relationship suggests that insight problem-solving could be related to, or modulated by, other factors that are highly affected by DA release, such as reward and feeling of pleasure. Aha! moments are indeed associated with a specific phenomenology characterized by pleasure, suddenness, feeling of certainty, and higher accuracy (Salvi et al., 2016a; Danek and Wiley, 2017; Danek and Carola, 2018; Laukkonen et al., 2020) and enhanced when rewarded by subliminal reward (Cristofori et al., 2018). In the same line, Oh et al. (2020) recorded high-density EEGs while participants solved a convergent thinking task and identified an insight-related effect that was modulated by reward sensitivity. Given recent neural evidence showing robust activation of the DA reward circuit when people experience having an Aha! moment (including the thalamic pathways, hippocampus, ventral tegmental area, nucleus accumbens, and substantia nigra; Tik et al., 2018), it may be that insight, in particular, recruits neural reward mechanisms related to the pathways that are impaired in PD patients and not restored by DRT. Unfortunately, only a few studies have examined creativity in PD patients empirically, and those which did it yielded mixed results and have never dealt with its association with insight. While our results do not allow us to specify what DA circuitry (if nigrostriatal or mesolimbic) is directly impaired by PD, the only conclusion we can draw is that insight problem-solving is disrupted in PD patients and cannot be restored by DRT.

Further, the conclusion that PD patients relate less to an insight style is also corroborated by the evidence that they make more errors of commission. Previous research on insight in general and on individual differences between more or less insightful people demonstrated that problem-solving via insight is associated with higher accuracy and more errors of omission (Smith and Kounios, 1996; Salvi et al., 2016a; Danek and Wiley, 2017; Webb et al., 2017; Danek and Carola, 2018; Laukkonen et al., 2020) whereas using a non-insight problem-solving style is seen to be associated with making more errors of commission (i.e., incorrect responses; Kounios et al., 2008; Salvi et al., 2016a). This effect is partially due to the all-or-nothing nature of Aha! moments, where solvers do not have access to intermediate knowledge while reasoning since it is processed below the solver's threshold of awareness, thus if the idea comes to mind it is either the correct solution or it does not come at all (Smith and Kounios, 1996; Kounios et al., 2008; Salvi et al., 2016a). As a result, insight problem-solving does not yield any intermediate results, and in the absence of meaningful potential solutions to guess with, those people who tend to rely on insight style more often time out rather than produce errors (Smith and Kounios, 1996). Conversely, step-by-step solving happens incrementally and above the threshold of awareness, allowing for partial information access on which a participant can base a guess just before the response deadline, hence a potential error of commission and lack of timeouts (Kounios et al., 2008; Salvi et al., 2016a). This notion explains why PD patients tend to make more errors of commission and fewer time-outs than controls because they rely less on insight and more on step-by-step problem-solving. These results further corroborate the idea that insight processing is impaired in PD patients, even when under DRT. Following the previous analysis, our result indicates that there might be a relation between the neurological impairment that affects PD patients and the tendency to generate ideas in a more step-by-step fashion. This allows us to draw two main conclusions: first, that the DA nigrostriatal pathway impairment in PD may also be involved in generating ideas *via* insight; and second, that PD patients may substitute insights by using a step-by-step problem-solving strategy which, since they are not processed by the basal ganglia, may remain unaltered by the disease state or DRT. In sum, while our results further confirm the involvement of the DA system in insight problem-solving, which in the former study was demonstrated only by correlation (Salvi et al., 2015a; Kizilirmak et al., 2016; Cristofori et al., 2018; Tik et al., 2018; Oh et al., 2020), it excludes its facilitation by DRT replacement. This leads to the hypothesis that this problem-solving style might originate in brain structures that are evolutionary more ancient (e.g., the basal ganglia) and processed by the cortex only in a second time (Laukkonen et al., 2020). While this explanation remains a theory, it is in line with recent literature theorizing the adaptive function of the Aha! feeling as a signal to more accurate ideas that are processed below awareness (Laukkonen et al., 2020).

In our hypothesis, we wondered if the association between PD and the rise of presumed creative “talent” represents an “awakening” or a “re-awakening” of creativity. In other words, we wanted to investigate the role of pre-PD creative dispositions in the rise of the creative “talent” observed in PD patients on DRT. From the literature on single cases, it appeared that most of the PD patients were already involved in some artistic activities before the diagnosis of PD, or were genetically related to highly creative people (Schrag and Trimble, 2001; Chatterjee et al., 2006; Walker et al., 2006). To better understand this relation, we administered to our sample scales to assess real-life creative achievement (CAQ and the HABPS). In regards to divergent thinking, our results support the idea that pre-existing real-life creative abilities do enhance some aspects of divergent thinking. Specifically, the creative achievement was the only one related to flexibility, while self-reported creativity as well as practicing a creative hobby for several years was related to fluency. The latter was also related to idea originality. Thus, it may be more likely that patients' engagement with creative activities, self-perceptions of creativity, or potential biological predispositions for artistic abilities are a necessary prelude to the improved divergent thinking observed for some PD patients under DRT. This result corroborates Canesi et al.'s (2016) finding that the Torrance Test for Adults for creative thinking was significantly higher for artists from both the healthy control and PD groups than other groups. Thus, the findings suggest that DRT causes a “reawakening” of creativity (specifically in divergent thinking flexibility, fluency, and originality), but only for PD patients who were already creative or engaged with creative hobbies before their PD diagnosis.

The results we obtained on convergent thinking and real-life creative predispositions are mixed across the different measures of CRA and Rebus Puzzles. Our results show a difference in trends for real-life creative achievements and self-reported creativity together with having a creative hobby. This is why “art-bias” might play a role. On one hand, we found that a history of creative achievements predicted enhanced convergent thinking on the Rebus Puzzles; but on the other hand, PD patients who perceived themselves as creative or had been practicing a creative hobby for many years displayed worse convergent thinking on the CRA, specifically in step-by-step solutions and not those via insight. This difference between Rebus Puzzles and CRAs indicates the potential for important task differences in how convergent thinking is measured and raises the question of whether some of the effects reported in past studies might be related to task differences.

Overall, the results indicate that pre-PD diagnosis creative achievements are a relevant predictor of creativity in both divergent and convergent thinking. Among our hypotheses, we also wondered if there is a misperception of “artistic-like” behaviors as creativity. Our results show that while creative achievements predict problem-solving accuracy, practicing a creative hobby or perceiving oneself as creative does not predict problem-solving accuracy for PD patients on DRT. We further explored how this misperception is particularly enhanced in PD patients who practice artistic hobbies, since these activities may contribute to perceiving themselves as creative. In conclusion, our results suggest it is likely that such an artistic production has been conflated with creativity in these patients, probably due to an “art-bias” where the creative label is often reserved only for artistic production (Runco, 2014).

Is the increase of creative talent followed by DRT in PD patients a compulsive reaction to the drugs, similar to hypersexuality, gambling, or punding caused by lack of inhibition? Does inhibition promote or impair creativity in PD patients when on DRT? From the current literature, it is unclear what role the lack of inhibition plays in the “artistic-like” behaviors that also emerge with DRT in these patients. It may be that the unstoppable art-production of the cases reported in the literature has little bearing on creativity, and may simply represent a compulsive behavior that provides a socially-accepted reward to patients.

Overall our results showed that executive functions are impaired in PD and that reduced inhibitory control in PD patients “on” DRT is associated with worse convergent thinking performance. Specifically, PD patient's lack of inhibition is negatively related to convergent thinking accuracy and positively with errors of commission. Interestingly, a better inhibitory control explained higher proportions of solutions *via* step-by-step. These results demonstrated that disrupted inhibitory control plays an important role in creativity and problem-solving, leading to more errors. Yet, better regulation does not seem to improve solutions *via* insight but a more aware step-by-step strategy. This result further strengthens our former suggestion that PD patients might rely more on an analytical problem-solving strategy in presence of cognitive control. This result is in line with the scientific literature showing a positive relationship between enhanced cognitive control and creativity (e.g., Benedek et al., 2014; Edl et al., 2014), especially for creative thinking involving insight (e.g., Mendelsohn, 1976; Gilhooly and Fioratou, 2009). Results reported by Benedek et al. (2014) support a relationship between executive abilities and creativity, as better working memory updating and inhibition scores predicted better performance. Other studies show that cognitive control (specifically measured by Stroop interference) is correlated with only certain aspects of creativity, arguing that better cognitive control may enhance creativity by suppressing dominant but irrelevant responses (Edl et al., 2014). The most important finding of our analysis is that the lack of inhibition negatively affects creativity (at least convergent thinking), and shows how PD patients with a stronger cognitive control are not just more accurate but also make fewer guesses (errors of commission) probably because they can better control the suppression of dominant, but irrelevant, ideas during the process of creative idea generation (Gilhooly et al., 2007; Benedek and Neubauer, 2013). This conclusion is in line with previous studies showing the executive nature of creative thoughts (Nusbaum and Silvia, 2011; Beaty and Silvia, 2012; Silvia and Beaty, 2012; Jauk et al., 2013, 2014). In conclusion, our results allow us to conclude that while the lack of inhibition caused by DRT may lead to compulsive artistic production, such a production is not translated into creativity.

While the results of this study allow us a better understanding of PD and the role of DRT in creativity we acknowledge some limitations, such as the small sample size. For our sample, we recruited PD patients who underwent two sessions of standard clinical assessment, one “on” and one “off” DRT medication before receiving deep brain stimulation surgery. Because of the cognitive and motor impairments of PD, these patients rarely go “off” medication and are difficult to recruit. To mitigate this limitation in clinical studies where small patient sample sizes are often unavoidable, statisticians have advocated reporting likelihood estimates such as Bayes factors (Lilford et al., 1995; Matthews, 1995; Billingham et al., 2012). In line with this recommendation, we reported Bayes factors for all relevant findings in the Results section. Critically, Bayes factors are relatively robust to small sample sizes and are therefore considered more reliable than traditional null-hypothesis-significance testing under such circumstances (Rouder et al., 2009; Van de Schoot et al., 2014). Given the lack of relevant pre-existing data for our main research questions, we used default priors to obtain our Bayes factor estimates; this practice is recommended for research questions for which little data exists, as it avoids biasing the calculations and uses only the current data to derive the Bayesian estimates (Billingham et al., 2012; Van de Schoot et al., 2014). Further, as reported in the Supplementary Material age, years of education, gender, disease duration, did not influence our results and their generalizability.

## Data Availability Statement

The raw data supporting the conclusions of this article will be made available by the authors, without undue reservation.

## Ethics Statement

The studies involving human participants were reviewed and approved by the ethical committee of the Area Vasta Emilia Nord. The patients/participants provided their written informed consent to participate in this study.

## Author Contributions

CS contributed in work coordination, conceptualization, methodology, data interpretation and analysis, and writing the original draft. EL contributed in data interpretation and analysis and writing. BB contributed in data collection and curation. MM contributed in work coordination, methodology, data collection, and clinical assessment. RE contributed in work coordination for PD recruitment and data collection. PN contributed in work coordination, conceptualization, methodology, and final editing. JG contributed in work supervision and coordination, conceptualization, and methodology. JD contributed in supervision, writing, reviewing, and final editing. All authors contributed to the article and approved the submitted version.

## Conflict of Interest

The authors declare that the research was conducted in the absence of any commercial or financial relationships that could be construed as a potential conflict of interest.

## Footnotes

^1^The same instructions used by Salvi et al. (2015a) were given to participants to explain how to distinguish solutions *via* insight from those via step-by-step. The original English instructions are: “[…] by INSIGHT means that the answer suddenly (i.e., unexpectedly) came to your mind, while you were trying to solve the problem, even though you are unable to articulate how you achieved the solution. This kind of solution is often associated with surprise exclamations such as ‘Aha!’; STEP-BY-STEP means that you figured out the answer after you deliberately and consciously tested out different words until you found the correct one. In this case for instance, you are able to report the steps that you used to reach the solution.”

^2^We are reporting Bayes Factor (BF_10_) for significant and null results. BF_10_ were computed in JASP (jasp-stats.org) using the default priors. We consider a BF_10_ of 3 or more to be analogous to a critical threshold, although BF_10_ can be interpreted continuously as the odds in favor of the alternative hypothesis. BF_10_ < 1 indicate more support for the null than the alternative hypothesis, and a BF_10_ < 1/3 would suggest moderate support for the null hypothesis (analogous to a BF_10_ of 3 in favor of the alternative hypothesis).

## References

[B1] Akbari Chermahini S. Hommel B. (2010). The (b)link between creativity and dopamine: spontaneous eye blink rates predict and dissociate divergent and convergent thinking. Cognition 115, 458–465. 10.1016/j.cognition.2010.03.007 20334856

[B2] Antonini A. Cilia R. (2009). Behavioral adverse effects of dopaminergic treatments in Parkinson's disease: incidence, neurobiological basis, management and prevention. Drug Safety 32, 475–488. 10.2165/00002018-200932060-00004 19459715

[B3] Aziz-Zadeh L. Kaplan J. T. Iacoboni M. (2009). “Aha!:” the neural correlates of verbal insight solutions. Human Brain Map. 30, 908–916. 10.1002/hbm.20554 18344174 PMC6870806

[B4] Beaty R. E. Benedek M. Silvia P. J. Schacter D. L. (2016). Creative cognition and brain network dynamics. Trends Cogn Sci. 20, 87–95. 10.1016/j.tics.2015.10.004 26553223 PMC4724474

[B5] Beaty R. E. Chen Q. Christensen A. P. Kenett Y. N. Silvia P. J. Benedek M.. (2020). Default network contributions to episodic and semantic processing during divergent creative thinking: a representational similarity analysis. NeuroImage 209:116499. 10.1016/j.neuroimage.2019.116499 31887423 PMC7056499

[B6] Beaty R. E. Kenett Y. N. Christensen A. P. Rosenberg M. D. Benedek M. Chen Q.. (2018). Robust prediction of individual creative ability from brain functional connectivity. Proc. Natl. Acad. Sci. U.S.A. 115, 1087–1092. 10.1073/pnas.1713532115 29339474 PMC5798342

[B7] Beaty R. E. Seli P. Schacter D. L. (2019). Network neuroscience of creative cognition: mapping cognitive mechanisms and individual differences in the creative brain. Curr. Opin. Behav. Sci. 27, 22–30. 10.1016/j.cobeha.2018.08.013 30906824 PMC6428436

[B8] Beaty R. E. Silvia P. J. (2012). Why do ideas get more creative across time? An executive interpretation of the serial order effect in divergent thinking tasks. Psychol. Aesthet. Creat. Arts 6, 309–319. 10.1037/a0029171

[B9] Becker M. Sommer T. Kühn S. (2020a). Verbal insight revisited: fMRI evidence for early processing in bilateral insulae for solutions with AHA! experience shortly after trial onset. Human Brain Map. 41, 30–45. 10.1002/hbm.24785 31520521 PMC7267914

[B10] Becker M. Wiedemann G. Kühn S. (2018). Quantifying insightful problem solving: a modified compound remote associates paradigm using lexical priming to parametrically modulate different sources of task difficulty. Psychol. Res. 84, 528–545. 10.31234/osf.io/cygwq 29951753

[B11] Becker M. Wiedemann G. Kühn S. (2020b). Quantifying insightful problem solving: a modified compound remote associates paradigm using lexical priming to parametrically modulate different sources of task difficulty. Psychol. Res. 84, 528–545. 10.1007/s00426-018-1042-3 29951753

[B12] Benedek M. Jauk E. Sommer M. Arendasy M. Neubauer A. C. (2014). Intelligence, creativity, and cognitive control: the common and differential involvement of executive functions in intelligence and creativity. Intelligence. 46, 73–83. 10.1016/j.intell.2014.05.007 25278640 PMC4175011

[B13] Benedek M. Neubauer A. C (2013). Revisiting Mednick's model on creativity-related differences in associative hierarchies. Evidence for a common path to uncommon thought. J. Creat. Behav. 47, 273–289. 10.1002/jocb.35 24532853 PMC3924568

[B14] Billingham L. Malottki K. Steven N. (2012). Small sample sizes in clinical trials: a statistician's perspective. Clin. Investig. 2, 655–657. 10.4155/cli.12.62

[B15] Blin O. Masson G. Azulay J. P. Fondarai J. Serratrice G. (1990). Apomorphine-induced blinking and yawning in healthy volunteers. Br. J. Clin. Pharmacol. 30, 769–773. 10.1111/j.1365-2125.1990.tb03848.x 2271377 PMC1368179

[B16] Bowden E. Jung-Beeman M. Fleck J. Kounios J. (2005). New approaches to demystifying insight. Trends Cogn. Sci. 9, 322–328. 10.1016/j.tics.2005.05.012 15953756

[B17] Bowden E. M. Jung-Beeman M. (2003). Normative data for 144 compound remote associate problems. Behav. Res. Methods Instr. Comp. 35, 634–639. 10.3758/BF03195543 14748508

[B18] Brück A. Aalto S. Nurmi E. Bergman J. Rinne J. O. (2005). Cortical 6-[18F]fluoro-L-dopa uptake and frontal cognitive functions in early Parkinson's disease. Neurobiol. Aging 26, 891–898. 10.1016/j.neurobiolaging.2004.07.014 15718048

[B19] Canesi M. Rusconi M. L. Cereda E. Ranghetti A. Cereda V. Moroni F.. (2017). Divergent thinking in parkinsonism: a case-control study. Front. Neurol. 8, 1–7. 10.3389/fneur.2017.00534 29118735 PMC5661018

[B20] Canesi M. Rusconi M. L. Isaias I. U. Pezzoli G. (2012). Artistic productivity and creative thinking in Parkinson's disease. Eur. J. Neurol. 19, 468–472. 10.1111/j.1468-1331.2011.03546.x 21981324

[B21] Canesi M. Rusconi M. L. Moroni F. Ranghetti A. Cereda E. Pezzoli G. (2016). Creative thinking, professional artists, and Parkinson's disease. J. Parkinson's Dis. 6, 239–246. 10.3233/JPD-150681 26639447

[B22] Carson S. H. Peterson J. B. Higgins D. M. (2005). Reliability, validity, and factor structure of the creative achievement questionnaire reliability, validity, and factor structure of the creative achievement questionnaire. Creativity Res. J. 14, 37–50. 10.1207/s15326934crj1701_4

[B23] Chakravarty A. (2010). The creative brain–revisiting concepts. Med. Hypotheses 74, 606–612. 10.1016/j.mehy.2009.10.014 19896776

[B24] Chatterjee A. Hamilton R. H. Amorapanth P. X. (2006). Art produced by a patient with Parkinson's disease. Behav. Neurol. Neurol. 17, 105–108. 10.1155/2006/901832 16873921 PMC5471527

[B25] Colzato L. S. Van Wouwe N. C. Hommel B. (2007). Spontaneous eyeblink rate predicts the strength of visuomotor binding. Neuropsychologia 45, 2387–2392. 10.1016/j.neuropsychologia.2007.03.004 17433381

[B26] Colzato L. S. van den Wildenberg W. P. M. van Wouwe N. C. Pannebakker M. M. Hommel B. (2009). Dopamine and inhibitory action control: evidence from spontaneous eye blink rates. Exp. Brain Res. 196, 467–474. 10.1007/s00221-009-1862-x 19484465 PMC2700244

[B27] Cools R. Barker R. A. Sahakian B. J. Robbins T. W. (2001a). Enhanced or imapired cognitive function in Parkinson's Disease as a function of dopaminergic medication and task demands. Cerebral Cortex 11, 1136–1143. 10.1093/cercor/11.12.1136 11709484

[B28] Cools R. Barker R. A. Sahakian B. J. Robbins T. W. (2001b). Mechanisms of cognitive set flexibility in Parkinson ' s disease. Brain 124, 2503–2512. 10.1093/brain/124.12.2503 11701603

[B29] Costa A. Bagoj E. Monaco M. Zabberoni S. De Rosa S. Papantonio A. M.. (2014). Standardization and normative data obtained in the Italian population for a new verbal fluency instrument, the phonemic/semantic alternate fluency test. Neurol. Sci. 35, 365–372. 10.1007/s10072-013-1520-8 23963806

[B30] Cristofori I. Salvi C. Beeman M. Grafman J. (2018). The effects of expected reward on creative problem solving. Cogn. Affect. Behav. Neurosci. 5, 925–931. 10.3758/s13415-018-0613-5 29949113 PMC6330050

[B31] Danek A. H. Carola S. (2018). Moment of truth: why Aha! experiences are correct. J. Creat. Behav. 54, 484–486. 10.1002/jocb.380 PMC13036825 41924231

[B32] Danek A. H. Wiley J. (2017). What about false insights? Deconstructing the Aha! experience along its multiple dimensions for correct and incorrect solutions separately. Front. Psychol. 7:2077. 10.3389/fpsyg.2016.02077 28163687 PMC5247466

[B33] Deumens R. Blokland A. Prickaerts J. (2002). Modeling Parkinson's disease in rats: an evaluation of 6-OHDA lesions of the nigrostriatal pathway. Exp. Neurol. 175, 303–317. 10.1006/exnr.2002.7891 12061862

[B34] Dubois B. Slachevsky A. Litvan I. Pillon B. (2000). The FAB: a frontal assessment battery at bedside. Neurology 55:1621–1626. 10.1212/WNL.55.11.1621 11113214

[B35] Edl S. Benedek M. Papousek I. Weiss E. M. Fink A. (2014). Creativity and the stroop interference effect. Pers. Ind. Diff. 69, 38–42. 10.1016/j.paid.2014.05.009

[B36] Eysenck H. J. (1993). Creativity and personality: suggestions for a theory. Psychol. Inquiry 4, 147–178. 10.1207/s15327965pli0403_1

[B37] Fasano A. Petrovic I. (2010). Insights into pathophysiology of punding reveal possible treatment strategies. Mol. Psychiatry 15, 560–573. 10.1038/mp.2009.95 20489735

[B38] Faust-Socher A. Kenett Y. N. Cohen O. S. Hassin-Baer S. Inzelberg R. (2014). Enhanced creative thinking under dopaminergic therapy in Parkinson disease. Ann. Neurol. 75, 935–942. 10.1002/ana.24181 24816898

[B39] Folstein Marshal F. Susan E. Folstein P. R. M. (1975). A practical state method for. J. Psychiatric Res. 12, 189–198. 10.1016/0022-3956(75)90026-6 1202204

[B40] Fossati A. Di Ceglie A. Acquarini E. Barratt E. S. (2001). Psychometric properties of an Italian version of the Barratt Impulsiveness Scale-11 (BIS-11) in nonclinical subjects. J. Clin. Psychol. 57, 815–828. 10.1002/jclp.1051 11344467

[B41] Friedman R. S. Förster J. (2005). Effects of motivational cues on perceptual asymmetry: implications for creativity and analytical problem solving. J. Personal. Soc. Psychol. 88, 263–275. 10.1037/0022-3514.88.2.263 15841858

[B42] Gilhooly K. J. Fioratou E. (2009). Executive functions in insight versus non-insight problem solving: an individual differences approach. Think. Reason. 15, 355–376. 10.1080/13546780903178615

[B43] Gilhooly K. J. Fioratou E. Anthony S. H. Wynn V. (2007). Divergent thinking: Strategies and executive involvement in generating novel uses for familiar objects. Br. J. Psychol. 98, 611–625. 10.1111/j.2044-8295.2007.tb00467.x 17535464

[B44] Grober E. Buschke H. (1987). Genuine memory deficits in dementia. Dev. Neuropsychol. 3, 13–16.

[B45] Gröger A. Kolb R. Schäfer R. Klose U. (2014). Dopamine reduction in the substantia nigra of Parkinson's disease patients confirmed by *in vivo* magnetic resonance spectroscopic imaging. PLoS ONE. 9:e84081. 10.1371/journal.pone.0084081 24416192 PMC3885536

[B46] Guilford J. P. (1967). The Nature of Human Intelligence. Vol. 5. New York, NY: McGraw Hill.

[B47] Hass R. W. Beaty R. E. (2018). Use or Consequences: probing the cognitive difference between two measures of divergent thinking. Front. Psychol. 9, 1–15. 10.3389/fpsyg.2018.02327 30542311 PMC6278612

[B48] Hass R. W. Rivera M. Silvia P. J. (2018). On the dependability and feasibility of layperson ratings of divergent thinking. Front. Psychol. 9, 1–13. 10.3389/fpsyg.2018.01343 30150952 PMC6099101

[B49] Heilman K. M. (2005). Creativity and the Brain. Psychology Press.

[B50] Henik A. Singh J. Beckley D. J. Rafal R. D. (1993). Disinhibition of automatic word reading in Parkinson's disease. Cortex 29, 589–599. 10.1016/S0010-9452(13)80283-3 8124936

[B51] Hsieh Y. H. Chen K. J. Wang C. C. Lai C. L. (2008). Cognitive and motor components of response speed in the Stroop test in Parkinson's disease patients. Kaohsiung J. Med. Sci. 24, 197–203. 10.1016/S1607-551X(08)70117-7 18424356 PMC11917918

[B52] Hughes A. J. Daniel S. E. Kilford L. Lees A. J. (1992). Accuracy of clinical diagnosis of idiopathic Parkinson's disease: a clinico-pathological study of 100 cases. J. Neurol. Neurosurg. Psychiatry. 55, 181–184. 10.1136/jnnp.55.3.181 1564476 PMC1014720

[B53] Inzelberg R. (2013). The awakening of artistic creativity and Parkinson's disease. Behav. Neurosci. 127, 256–261. 10.1037/a0031052 23316709

[B54] Jauk E. Benedek M. Dunst B. Neubauer A. C. (2013). The relationship between intelligence and creativity: New support for the threshold hypothesis by means of empirical breakpoint detection. Intelligence 41, 212–221. 10.1016/j.intell.2013.03.003 23825884 PMC3682183

[B55] Jauk E. Benedek M. Neubauer A. C. (2014). Theroadtocreativeachievement: a latent variablemodel ofability and personality predictors. Euro. J. Pers. 28, 95–105. 10.1002/per.1941 24532953 PMC3923982

[B56] Jung-Beeman M. Bowden E. M. Haberman J. Frymiare J. L. Arambel-Liu S. Greenblatt R.. (2004). Neural activity when people solve verbal problems with insight. PLoS Biol. 2, 500–510. 10.1371/journal.pbio.0020097 15094802 PMC387268

[B57] Karson C. N. (1983). Spontaneous eye-blink rates and dopaminergic systems. Brain 106, 643–653. 10.1093/brain/106.3.643 6640274

[B58] Kizilirmak J. M. Thuerich H. Folta-Schoofs K. Schott B. H. (2016). Neural correlates of learning from induced insight: a case for reward-based episodic encoding. Front. Psychol. 7, 1–16. 10.3389/fpsyg.2016.01693 27847490 PMC5088210

[B59] Kleven M. S. Koek W. (1996). Differential effects of direct and indirect dopamine agonists on eye blink rate in cynomolgus monkeys. J. Pharmacol. Exp. Therapeut. 279, 1211–1219. 8968343

[B60] Kounios J. Fleck J. I. Green D. L. Payne L. Stevenson J. L. Bowden E. M.. (2008). The origins of insight in resting-state brain activity. Neuropsychologia 46, 281–291. 10.1016/j.neuropsychologia.2007.07.013 17765273 PMC2293274

[B61] Kounios J. Frymiare J. L. Bowden E. M. Fleck J. I. Subramaniam K. Parrish T. B.. (2006). The prepared mind: neural activity prior to problem presentation predicts subsequent solution by sudden insight. Psychol. Sci. 17, 882–890. 10.1111/j.1467-9280.2006.01798.x 17100789

[B62] Kulisevsky J. Pagonabarraga J. Martinez-Corral M. (2009). Changes in artistic style and behaviour in Parkinson's disease: dopamine and creativity. J. Neurol. 256, 816–819. 10.1007/s00415-009-5001-1 19240966

[B63] Laukkonen R. Webb M. E. Salvi C. Tangen J. M. Schooler J. (2018). Eureka Heuristics: How feelings of insight signal the quality of a new idea. PsyArXiv. 10.31234/osf.io/ez3tn

[B64] Laukkonen R. E. Webb M. E. Salvi C. Schooler J. W. Tangen J. M. (2020). The Eureka heuristic: relying on insight to appraise the quality of ideas. [Preprint]. 1−44.

[B65] Lhommée E. Batir A. Quesada J. Ardouin C. Fraix V. Seigneuret E.. (2014). Dopamine and the biology of creativity: lessons from Parkinson's disease. Front. Neurol. 5:55. 10.3389/fneur.2014.00055 24795692 PMC4001035

[B66] Lilford R. J. Thornton J. G. Braunholtz D. (1995). Clinical trials and rare diseases: a way out of a conundrum. BMJ 311, 1621–1625. 10.1136/bmj.311.7020.1621 8555809 PMC2551510

[B67] Ludwig A. M. (1992). Creative achievement and psychopathology: comparison among professions. Am. J. Psychotherapy 46, 330–356. 10.1176/appi.psychotherapy.1992.46.3.330 1530096

[B68] Lumme V. Aalto S. Ilonen T. Någren K. Hietala J. (2007). Dopamine D2/D3 receptor binding in the anterior cingulate cortex and executive functioning. Psychiatry Res. Neuroimaging 156, 69–74. 10.1016/j.pscychresns.2006.12.012 17683918

[B69] MacGregor J. N. Cunningham J. B. (2008). Rebus puzzles as insight problems. Behav. Res. Methods 40, 263–268. 10.3758/BRM.40.1.263 18411549

[B70] Matthews J. N. S. (1995). Small clinical trials: are they all bad? Statist. Med. 14, 115–126. 10.1002/sim.4780140204 7754260

[B71] Mednick S. A. (1968). Remote associates test. J. Creative Behav. 2, 213–214. 10.1002/j.2162-6057.1968.tb00104.x

[B72] Mendelsohn G. A. (1976). Associative and attentional processes in creative performance. J. Personality. 44, 341–369. 10.1111/j.1467-6494.1976.tb00127.x

[B73] Milgram R. M. Milgram N. A. (1976). Creative thinking and creative performance in Israeli students. J. Educ. Psychol. 68, 255–259. 10.1037/0022-0663.68.3.255 932305

[B74] Nusbaum E. C. Silvia P. J. (2011). Are intelligence and creativity really so different? Fluid intelligence, executive processes, and strategy use in divergent thinking. Intelligence 39, 36–45. 10.1016/j.intell.2010.11.002

[B75] Oh Y. Chesebrough C. Erickson B. Zhang F. Kounios J. (2020). An insight-related neural reward signal. NeuroImage 214:116757. 10.1016/j.neuroimage.2020.116757 32194279

[B76] Patton J. H. Stanford M. S. Barratt E. S. (1995). Factor structure of the barratt impulsiveness scale. J. Clin. Psychol. 51, 768–774. 10.1002/1097-4679(199511)51:6<768::AID-JCLP2270510607>3.0.CO;2-1 8778124

[B77] Preacher K. J. (2002). Calculation for the Test of the Difference Between Two Independent Correlation Coefficients (Computer software). Available online at: http://quantpsy.org

[B78] Raven J. C. (1965). Advanced Progressive Matrices: Sets I and II: Plan and Use of the Scale With a Report of Experimental Work Carried Out by G. A. Foulds and A. R. Forbes. London: H. K. Lewis.

[B79] Rouder J. N. Speckman P. L. Sun D. Morey R. D. Iverson G. (2009). Bayesian *t*-tests for accepting and rejecting the null hypothesis. Psychon. Bull. Rev. 16, 225–237. 10.3758/PBR.16.2.225 19293088

[B80] Runco M. A. (2014). Creativity: Theories and Themes: Research, Development, and Practice. Elsevier.

[B81] Salvi C. Beeman M. Bikson M. McKinley R. Grafman J. (2020a). TDCS to the right anterior temporal lobe facilitates insight problem-solving. Sci. Rep. 10:946. 10.1038/s41598-020-57724-1 31969588 PMC6976642

[B82] Salvi C. Bowden E. (2019). The relation between state and trait risk taking and problem-solving. Psychol. Res. 84, 1235–148. 10.1007/s00426-019-01152-y 30756178 PMC6690799

[B83] Salvi C. Bricolo E. Franconeri S. L. Kounios J. Beeman M. (2015a). Sudden insight is associated with shutting out visual inputs. Psychon. Bull. Rev. 22, 1814–1819. 10.3758/s13423-015-0845-0 26268431

[B84] Salvi C. Bricolo E. Kounios J. Bowden E. Beeman M. (2016a). Insight solutions are correct more often than analytic solutions. Think. Reason. 22, 1–18. 10.1080/13546783.2016.1141798 27667960 PMC5035115

[B85] Salvi C. Costantini G. Bricolo E. Perugini M. Beeman M. (2015b). Validation of Italian rebus puzzles and compound remote associate problems. Behav. Res. Methods. 48, 664–685. 10.3758/s13428-015-0597-9 26148823

[B86] Salvi C. Costantini G. Pace A. Palmiero M. (2018). Validation of the Italian remote associate test. J. Creat. Behav. 1–13. 10.1002/jocb.345 32728265 PMC7388808

[B87] Salvi C. Cristofori I. Grafman J. Beeman M. (2016b). The politics of insight. Quart. J. Exp. Psychol. 69, 1064–1072. 10.1080/17470218.2015.1136338 26810954 PMC4869693

[B88] Salvi C. Iannello P. Cancer A. MacClay M. Rago S. Dunsmoor J. E.. (2020b). Going viral: how fear, socio-cognitive polarization and problem-solving influence fake news detection and proliferation during COVID-19 pandemic. Front. Commun. 5, 1–16. 10.3389/fcomm.2020.562588

[B89] Salvi C. Simoncini C. Grafman J. Beeman M. (2020c). Oculometric signature of switch into awareness? Pupil size predicts sudden insight whereas microsaccades predict problem-solving via analysis. NeuroImage 217:116933. 10.1016/j.neuroimage.2020.116933 32413459 PMC7440842

[B90] Santarnecchi E. Sprugnoli G. Bricolo E. Constantini G. Liew S. L. Musaeus C. S.. (2019). Gamma tACS over the temporal lobe increases the occurrence of Eureka! moments. Sci. Rep. 9:5778. 10.1038/s41598-019-42192-z 30962465 PMC6453961

[B91] Schrag A. Trimble M. (2001). Poetic talent unmasked by treatment of Parkinson's disease Anette. Mov. Disord. 16, 1171–1174. 10.1002/mds.1239 11748756

[B92] Scott G. M. Lonergan D. C. Mumford M. D. (2005). Reliability, validity, and factor structure of the creative achievement questionnaire. Creativ. Res. J. 17, 79–98. 10.1207/s15326934crj1701_7

[B93] Silvia P. J. Beaty R. E. (2012). Making creative metaphors: the importance of fluid intelligence for creative thought. Intelligence 40, 343–351. 10.1016/j.intell.2012.02.005

[B94] Smith R. W. Kounios J. (1996). Sudden insight: all-or-none processing revealed by speed-accuracy decomposition. J. Exp. Psychol. Learn. Memory Cogn. 22, 1443–1462. 10.1037/0278-7393.22.6.1443 8921602

[B95] Sprugnoli G. Liew S. L. Bricolo E. Costantini G. Salvi C. Musaeus C. S.. (2017). Going beyond the Eureka moment: enhancement of insightful solutions by means of tACS and tRNS. Brain Stimul. 10:402.

[B96] Sternberg R. J. Lubart T. I. (1996). An investment perspective creative insight on, in The Nature of Insight, eds Sternberg R. J. Davidson J. E. (The MIT Press), 535–558.

[B97] Stroop J. R. (1935). Studies of interference in serial verbal reactions. J. Exp. Psychol. 121, 15–23. 10.1037/0096-3445.121.1.15

[B98] Subramaniam K. Kounios J. Parrish T. B. Jung-Beeman M. (2009). A brain mechanism for facilitation of insight by positive affect. J. Cogn. Neurosci. 21, 415–432. 10.1162/jocn.2009.21057 18578603

[B99] Taylor J. R. Elsworth J. D. Lawrence M. S. Sladek J. R. Roth R. H. Redmond D. E. (1999). Spontaneous blink rates correlate with dopamine levels in the caudate nucleus of MPTP-treated monkeys. Exp. Neurol. 158, 214–220. 10.1006/exnr.1999.7093 10448434

[B100] Threadgold E. Marsh J. E. Ball L. J. (2018). Normative data for 84 UK English rebus puzzles. Front. Psychol. 9:2513. 10.3389/fpsyg.2018.02513 30618942 PMC6300574

[B101] Threadgold E. Marsh J. E. McLatchie N. Ball L. J. (2019). Background music stints creativity: Evidence from compound remote associate tasks. Appl. Cogn. Psychol. 33, 873–888. 10.1002/acp.3532

[B102] Tik M. Sladky R. Luft C. D. B. Willinger D. Hoffmann A. Banissy M. J.. (2018). Ultra-high-field fMRI insights on insight: neural correlates of the Aha!-moment. Human Brain Mapp. 39, 3241–3252. 10.1002/hbm.24073 29665228 PMC6055807

[B103] Torrance E. P. (1974). The Torrance Tests of Creative Thinking-Norms-Technical Manual Research Edition-Verbal Tests, Forms A and B- Figural Tests, Forms A and B. Princeton, NJ: Personnel Press.

[B104] Tune L. Barta P. Wong D. Powers R. E. Pearlson G. Tien A. Y.. (1996). Striatal dopamine D2 receptor quantification and superior temporal gyrus: volume determination in 14 chronic schizophrenic subjects. Psychiatry Res. 67, 155–158. 10.1016/0925-4927(96)02728-X 8876015

[B105] Van de Schoot R. Kaplan D. Denissen J. Asendorpf J. B. Neyer F. J. van Aken M. A. G. (2014). A gentle introduction to Bayesian analysis: applications to developmental research. Child Dev. 85, 842–860. 10.1111/cdev.12169 24116396 PMC4158865

[B106] Walker R. H. Warwick R. Cercy S. P. (2006). Augmentation of artistic productivity in Parkinson's disease. Mov. Disord. 21, 285–286. 10.1002/mds.20758 16261619

[B107] Webb M. E. Little D. R. Cropper S. J. (2017). Once more with feeling: normative data for the aha experience in insight and noninsight problems. Behav. Res. Methods 50, 2035–2056. 10.3758/s13428-017-0972-9 29052169

[B108] Wechsler Abbreviated Scale of Intelligence (1999). Manual. San Antonio, TX: The Psychological Corporation.

[B109] Weintraub D. Mamikonyan E. Papay K. Judith A. Xie S. X. Siderowf A. (2012). Questionnaire for impulsive-compulsive disorders in Parkinson's disease–rating scale. Mov. Disord. 27, 242–247. 10.1002/mds.24023 22134954 PMC3537263

