# Supplementary material for: The Effect of Dopaminergic Replacement Therapy on Creative Thinking and Insight Problem-Solving in Parkinson's Disease Patients
Source: Front Psychol. 2021 Mar 5;12:646448. doi: 10.3389/fpsyg.2021.646448 (PMC7984162; doi:10.3389/fpsyg.2021.646448)
Supplement: Supplementary file 1 [file Data_Sheet_1.pdf]

Supplemental Results

Potential Confounds

Demographic Characteristics. PD patients and HC participants did not significantly differ in age, gender composition, or education (see Supplemental Table 1 for relevant statistics). PD patients in the two orders (ON-OFF, OFF-ON) did not significantly differ on any of the above demographic variables nor any clinical variables (all BF_{10}=0.46 to 1.43), including years since PD diagnosis, impulsivity or impulsive-compulsive disorder behavior (BIS, QUIP-ICD, and RS scores), punding (HABPS score), general mental status (MMSE score), cognitive flexibility (RPM score), language abilities (VFT-P and VFT-S scores), memory (FCSRT scores), or executive function (FAB, ST-EIE, ST-EIT, DS-F, and DS-B scores). All participants scored ≥ 24 on the MMSE, indicating no evidence of cognitive impairment or dementia within our sample.

Order and Practice Effects. To evaluate the potential interaction of ON/OFF order and DRT status in PD patients, we conducted a series of paired sample *t*-tests comparing within-subject DRT effects on creative thinking performance between the two orders (ON-OFF, OFF-ON) for the AUT, CRA, and Rebus Puzzles.

Slowed Processing and Motor Functioning. Although we attempted to mitigate the influence of motor deficiencies on behavioral performance by having the Experimenter enter the responses for PD patients on the divergent and convergent thinking tasks, there may have remained some confounding influence of cognitive or motor slowing on our main findings that could not be avoided (for example if PD patients were slow to communicate when a solution was reached, leading to more timeouts). We, therefore, sought to assess the degree to which PD patients' reduced performance on the CRA and Rebus Puzzles reflected potential confounds of slowed processing or motor function, two prominent symptoms of PD which could hinder the ability to generate solutions within the allotted time. To that end, we used independent sample *t*-tests to compare

the rate of timeouts on the CRA and Rebus Puzzles between PD patients 'on' DRT and HCs. This showed that PD patients 'on' DRT experienced significantly fewer timeouts than HCs on the CRA (t_{35}=2.26, *p*=.030, 95% CI=[.012, .227], Cohen's *d*=0.78), but did not significantly differ from HCs in the rate of timeouts on the Rebus Puzzles. Thus, there was no statistical evidence that PD patients experienced more timeouts on the CRA or Rebus Puzzles than HC participants, contrary to what might be expected if reduced processing speed or motor function interfered with task performance for the PD group.

As a further check on the potential relationship between speed and convergent thinking performance, we examined the Pearson correlations of timeouts and error rates on the CRA and Rebus Puzzles for both PD patients 'on' DRT and HCs. On the CRA, error rates and timeouts were significantly negatively correlated for PD patients 'on' DRT (*r*=-.61, *p*=.027; 95% CI=[-.87,-.09]) and HCs (*r*=-.82, *p*<.001; 95% CI=[-.92, -.62]). Also, error rates and timeouts on the Rebus Puzzles were negatively correlated for HC participants (*r*=-.60, *p*=.002; 95% CI=[-.81, -.02]), although they did not correlate for PD patients 'on' DRT. Combined with the findings from the main analyses that reduced convergent thinking performance was related to greater Stroop interference (and thus poorer cognitive inhibition), these inverse associations suggest that the reduced accuracy of PD patients may not have been a mere reflection of slowed cognitive processing or motor function, but instead may reflect impaired inhibition or executive function.

Executive Function. Given we could not find statistical evidence that PD patients' higher error rate on the convergent thinking tasks reflected slowed processing or motor function, we next sought to assess if the increased error rate might reflect impaired executive functions—another prominent feature of PD. To obtain an initial overview of executive function differences between PD patients and HCs, we first conducted a series of independent sample *t*-tests comparing scores between the two groups on a range of assessments of executive function, including the MMSE, RPM, FAB, and DS-F and DS-B. Although Stroop performance is also considered to assess executive function, given its particular relevance for cognitive

inhibition it was reported in an earlier section (see *Inhibition and Creative Thinking Behavior*) and is not included here. In cases where the equal variance assumption was violated (i.e., for the MMSE and FAB) results are from Welch's *t*-tests; otherwise, results are from Student's *t*-tests. In general, PD patients showed reduced executive function compared to HCs, scoring significantly lower than HCs on the MMSE (Welch's $t_{16.42}$=-2.51, p=.047; 95% CI=[-1.94, -0.02]; Cohen's d=-0.79), RPM (t_{35}=-3.53, p=.001; 95% CI=[-6.93, -1.87]; Cohen's d=-1.22), and FAB (Welch's $t_{13.45}$=-2.84, p=.014; 95% CI=[-1.90, -0.26]; Cohen's d=-1.09). No other significant effects emerged.

Next, we assessed the extent to which executive function is related to error rates on the convergent thinking measures. To that end, we examined the Pearson correlations between the above executive function measures and error rates on the CRA and Rebus Puzzles separately for PD patients 'on' DRT and Controls. No significant correlations between executive function and convergent thinking errors emerged for either group. Thus, there was no statistical evidence that the higher error rates in convergent thinking for PD patients *versus* HCs were related to reduced executive function. Taken together with the above findings, this suggests that the reduced convergent thinking performance of PD patients compared to HCs may not have simply reflected impairments in executive function.

Normality of Data for Linear Regression Findings. To verify that the residuals for the significant linear regression findings reported in the main text were normally distributed, we report normal probability plots (i.e., QQ-plots) of the standardized residuals against theoretical quantiles of the normal distribution for the associated regression models. QQ-plots are the advised method to assess normality when the sample size is limited, as formal normality tests and skewness-kurtosis statistics perform unreliably in small samples. As shown in the Q-Q plots (provided in Supplemental

Figures 1-3), the residuals from a reasonably close linear approximation and show no large departures from normality or pronounced fanning (factors which might contraindicate the use of linear regression if present). This suggests that the normality assumption was satisfied for the reported linear regression findings reported in the main text.

Supplemental Figure 1.

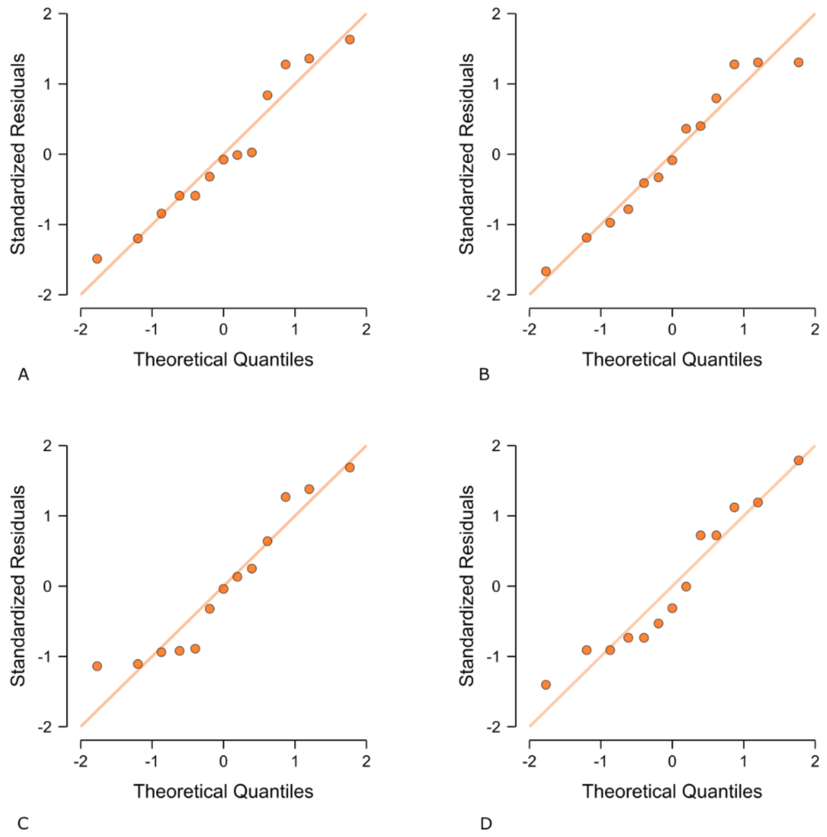

Supplemental Figure 1. QQ-plots for the significant regression models of real-life creativity predicting effects of DRT on divergent thinking in PD patients 'on' medication. Plots show standardized residuals reasonably approximate the theoretical quantiles of the normal distribution for the significant regressions for A. CAQ and flexibility; B. Self-reported creativity and fluency; C. Years of creative hobbies and originality; and D. Years of creative hobbies and fluency.

Note. CAQ=Creative Achievement Questionnaire.

Supplemental Figure 2.

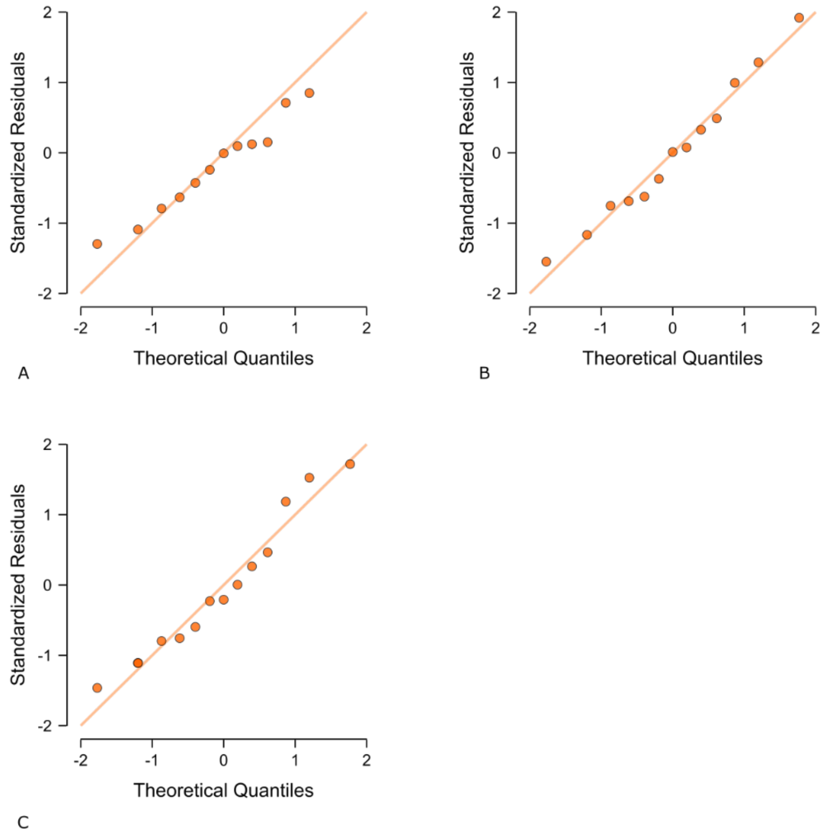

Supplemental Figure 2. QQ-plots for the significant regression models of real-life creativity predicting effects of DRT on convergent thinking for PD patients 'on'-'off' medication. Plots show standardized residuals reasonably approximate the theoretical quantiles of the normal distribution for the significant regressions for A. CAQ and Rebus accuracy; B. Self-reported creativity and CRA accuracy; and C. Years of creative hobbies and CRA solutions via step-by-step.

Note. CAQ=Creative Achievement Questionnaire. CRA=Compound Remote Associates Task.

Supplemental Figure 3.

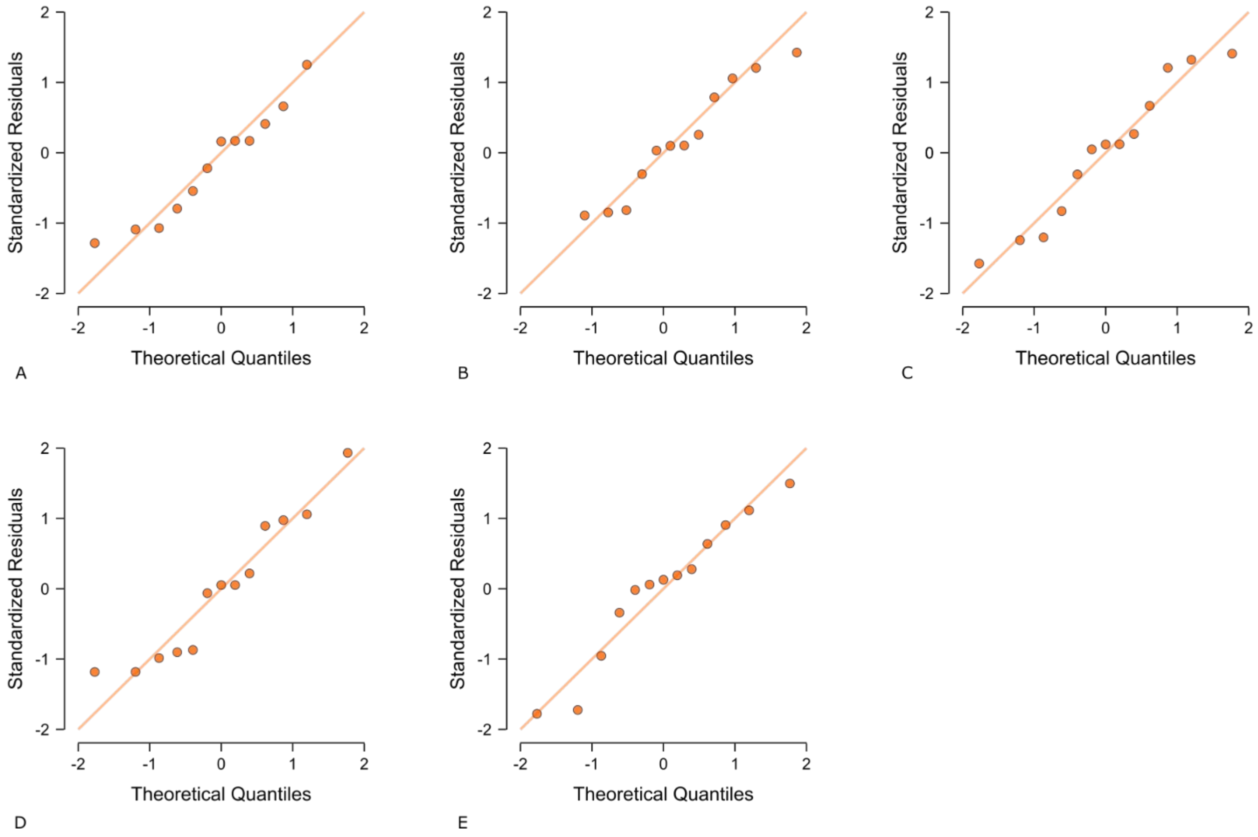

Supplemental Figure 3. QQ-plots for the significant regression models of Stroop interference effects predicting effects of DRT on convergent thinking for PD patients 'on' medication. Plots show standardized residuals reasonably approximate the theoretical quantiles of the normal distribution for the significant regressions for A. Stroop-EIE and CRA accuracy; B. Stroop-EIT and CRA commission errors; C. Stroop-EIT and CRA solutions via step-by-step; D. Stroop-EIE and Rebus accuracy; and E. Stroop-EIT and Rebus accuracy.

Note. CRA=Compound Remote Associates Task. Stroop-EIE=Stroop-Effect Interference Error. Stroop-EIT=Stroop-Effect Interference Time.

Supplemental Table 1

Descriptive Statistics for Demographic, Clinical, and Questionnaire Data for PD patients compared to HCs.

	PD		Controls							
	M	SD	M	SD	*t*	*df*	*p*	*95% CI*	*Cohen's d*	BF_{10}
Demographics										
Age, years	56.5	9.0	61.5	7.4	-1.84	35	.075	[-10.61, 0.54]	-0.63	1.18
Education, years	12.6	3.8	14.1	2.8	-1.33	35	.192	[-3.71, 0.77]	-0.46	0.65
Sex, M:F	9:4		11:13		-1.36	35	.182	[-0.58, 0.12]	-0.47	0.67
Duration PD, years	8.7	3.9								
Clinical Assessments										
Mental State (Dementia Screen) MMSE †	**27.3**	1.5	**28.3**	0.9	**-2.51**	16.42	**.047**	[-1.94, -0.02]	-0.79	**3.41**
Cognitive Flexibility: RPM	**28.5**	4.3	**32.9**	3.2	**-3.53**	35	**.001**	[-6.93, -1.87]	-1.26	**26.97**
General Executive Function: FAB †	**16.2**	1.3	**17.3**	0.4	**-2.84**	13.45	**.014**	[-1.90, -0.26]	-1.09	**35.49**
Memory Recall- Immediate: FCSRT-ITR	**45.2**	1.9	**46.6**	1.7	**-2.31**	35	**.027**	[-2.62, -0.17]	-0.48	**2.39**
Memory Recall- Delayed: FCSRT-DTR †	15.1	2.5	15.8	0.4	-1.02	12.36	.326	[-2.23, 0.80]	-0.40	0.68
Verbal Fluency- Letter: VFT-F	**35.4**	10.2	**42.7**	7.7	**-2.43**	35	**.020**	[-13.29, -1.20]	-1.41	**2.96**
Verbal Fluency- Semantic: VFT-S	**45.7**	7.9	**56.3**	7.3	**-4.10**	35	**<.001**	[-15.90, -5.37]	0.70	**103.41**
Cognitive Inhibition- Error Rate: ST-EIE †	0.9	1.0	0.3	0.6	1.74	16.15	.102	[-0.12, 1.20]	0.64	1.58
Cognitive Inhibition- Delay: ST-EIT †	20.5	11.2	18.7	4.9	0.52	14.55	.608	[-5.27, 8.69]	0.20	0.39
Attention: DS-F	8.5	1.4	9.0	1.4	-1.10	35	.281	[-1.54, 0.46]	-0.60	0.52
Working Memory: DS-B	7.5	1.9	8.6	2.0	-1.75	35	.089	[-2.52, 0.19]	0.26	1.05
Impulsivity: BIS	61.7	5.4	60.3	5.5	0.74	35	.462	[-2.42, 5.22]	0.43	0.41

Impulsive-Compulsive Behaviors: QUIP-ICD	11.1	7.3	8.3	5.8	1.26	35	.216	[-1.68, 7.17]	0.75	0.61
Impulsive-Compulsive Behavior Severity: QUIP-RS †	22.2	14.7	13.8	8.8	1.88	16.80	.078	[-1.05, 17.77]	0.69	1.92
Punding: HABPS	35.1	9.7	30.6	9.8	1.33	35	.196	[-2.37, 11.27]	-0.23	0.65
Real-World Creativity										
Creative Achievements: CAQ [a]	7.5	5.1	11.1	19.0	-0.70	35	.501	[-3.92, 3.98]	0.01	0.39
Years of Creative Hobby: HABPS - Item 2	12.8	12.6	20.2	14.3	-1.57	35	.125	[-17.04, 2.16]	-0.54	0.85
Self-Reported Creativity: HABPS - Session 3	6.5	2.5	4.9	2.6	1.77	35	.086	[-0.24, 3.41]	0.61	1.08

Note. MMSE=Mini-Mental State Examination RPM=Raven's Progressive Matrices FAB=Frontal Assessment Battery FCSRT=Free and Cued Selective Recall Test subscores (ITR=Immediate Total Recall; DTR=Delayed Total Recall); VFT=Verbal Fluency Test subscores (F=Letter; S=Semantic Category); ST=Stroop Test scores (EIE=Effect Interference Error; EIT=Effect Interference Time); BIS=Behavioral Inhibition Scale QUIP=Questionnaire for Impulsive-Compulsive Disorders in Parkinson's Disease total scores (ICD=Impulse Control Disorders, Full Version; RS=Rating Scale, Abbreviated Version); HABPS=Hobbyism and Artistic-like Behaviours Punding Scale; CAQ=Creative Achievement Questionnaire; HABPS Item-2: item 2 on the HABPS assessing the number of years devoted to the hobby; HABPS -Session 3: the session 3 subscores on the HABPS assessing self-reported creativity. Significant differences at $p < .05$ (two-tailed) and associated Bayes factor (BF_{10}) are bolded.

[a] One HC participant had a CAQ score that far exceeded 2 *SD*s + *M* and was treated as an outlier (excluded) from all analyses involving CAQ. The resulting HC group summary statistics for CAQ after excluding this participant were *M*=7.5, *SD*=5.9.

† indicates usage of Welch's *t*-test due to unequal variances between PD patients 'on' medication and HCs; all others were Student's *t*-tests.

Supplemental Table 2

Divergent Thinking in PD Patients 'On' DRT on versus 'Off' DRT and HCs.

Divergent Thinking	PD				HCs		Group Comparison: PD 'On' DRT *vs* HCs				
	'Off' DRT		'On' DRT								
	M	*SD*	*M*	*SD*	*M*	*SD*	*t*(35)	*p*	95% CI	Cohen's *d*	BF_{10}
AUT											
Fluency	8.26	3.09	8.28	2.15	9.55	2.31	-1.63	.111	[-2.85, 0.31]	-0.56	0.91
Flexibility	3.64	1.76	**4.85**	2.49	**8.58**	2.11	**-4.81**	**<.001**	[-5.30, -2.16]	-1.66	**626.13**
Elaboration	6.98	2.90	7.15	1.92	6.89	2.47	0.33	.741	[-1.34, 1.87]	0.12	0.34
Originality	12.52	6.78	12.97	5.17	16.35	6.61	-1.60	.120	[-7.68, 0.92]	-0.55	0.87

Note. Divergent thinking performance was indexed as AUT subscores for fluency, flexibility, elaboration, and originality. Significant differences between PD patients 'On' DRT and HCs at $p < .05$ (two-tailed, Student's *t*-test) and associated Bayes factor (BF_{10}) are bolded. No significant differences for PD patients 'On' *versus* 'Off' DRT were found.

Supplemental Table 3

Convergent Thinking in PD Patients 'On' DRT on versus 'Off' DRT and HCs.

Convergent Thinking	PD				HCs		Group Comparison: PD 'On' DRT *vs* HCs				
	'Off' DRT		'On' DRT								
	M	*SD*	*M*	*SD*	*M*	*SD*	$t^{\dagger}$	*p*	95% CI	Cohen's *d*	BF_{10}
CRA											
Accuracy	.31	.14	.34	.15	.40	.09	-1.28	.219	[-0.15, 0.04]	-0.47	0.76
Commission Errors	.41	.21	**.39**	.19	**.22**	.13	**3.34**	**.002**	[0.07, 0.28]	1.15	**17.47**
Omission Errors	.27	.15	**.26**	.14	**.38**	.16	**-2.26**	**.030**	[-0.23, -0.01]	-0.78	**2.23**
Insight	.19	.12	**.20**	.12	**.32**	.10	**-3.39**	**.002**	[-0.20, -0.05]	-1.17	**19.67**
Step-by-Step	.12	.16	.15	.15	.08	.08	1.51	.149	[-0.03, 0.16]	0.56	1.09
Rebus Puzzles											
Accuracy	.53	.22	**.52**	.12	**.70**	.08	**-5.72**	**<.001**	[-0.25, -0.12]	-1.97	**7075.82**
Commission Errors	.33	.17	**.34**	.08	**.17**	.08	**6.21**	**<.001**	[0.11, 0.23]	2.14	**26589.50**
Omission Errors	.12	.09	.13	.07	.11	.06	0.89	.380	[-0.03, 0.07]	0.31	0.45
Insight	.39	.26	**.38**	.13	**.57**	.11	**-4.84**	**<.001**	[-0.27, -0.11]	-1.67	**670.56**
Step-by-Step	.15	.20	.14	.11	.13	.07	0.20	.845	[-0.06, 0.07]	0.07	0.34

Note. Convergent thinking is indexed as behavioral response rates (out of the total number of trials given) on the CRA and Rebus Puzzles for accuracy (proportion correct), commission errors (proportion incorrect), omission errors (proportion where time expired before a solution was reached), and solutions generated via insight and step-by-step problem-solving styles (proportion solved via each method). Significant differences between PD patients 'On' DRT and HCs at $p < .05$ (two-tailed) and associated Bayes factor (BF_{10}) are bolded. No significant differences for PD patients 'On' *versus* 'Off' DRT were found.

† Accuracy and step-by-step solution rates for PD patients 'on' DRT *versus* HCs were compared using Welch's *t*-test (*df*=16.74 and 16.28, respectively) due to violation of the equal variance assumption; all others were Student's *t*-tests (*df*=35).
